# Modifications and Trafficking of APP in the Pathogenesis of Alzheimer’s Disease

**DOI:** 10.3389/fnmol.2017.00294

**Published:** 2017-09-15

**Authors:** Xin Wang, Xuan Zhou, Gongying Li, Yun Zhang, Yili Wu, Weihong Song

**Affiliations:** ^1^Department of Psychiatry, Jining Medical University Jining, China; ^2^Shandong Key Laboratory of Behavioral Medicine, Jining Medical University Jining, China; ^3^Collaborative Innovation Center for Birth Defect Research and Transformation of Shandong Province, Jining Medical University Jining, China; ^4^Townsend Family Laboratories, Department of Psychiatry, The University of British Columbia Vancouver, BC, Canada

**Keywords:** Alzheimer’s disease, APP, Aβ, post-translational modifications, trafficking

## Abstract

Alzheimer’s disease (AD), the most common neurodegenerative disorder, is the leading cause of dementia. Neuritic plaque, one of the major characteristics of AD neuropathology, mainly consists of amyloid β (Aβ) protein. Aβ is derived from amyloid precursor protein (APP) by sequential cleavages of β- and γ-secretase. Although APP upregulation can promote AD pathogenesis by facilitating Aβ production, growing evidence indicates that aberrant post-translational modifications and trafficking of APP play a pivotal role in AD pathogenesis by dysregulating APP processing and Aβ generation. In this report, we reviewed the current knowledge of APP modifications and trafficking as well as their role in APP processing. More importantly, we discussed the effect of aberrant APP modifications and trafficking on Aβ generation and the underlying mechanisms, which may provide novel strategies for drug development in AD.

## Introduction

Alzheimer’s disease (AD), the most common neurodegenerative disorder leading to dementia, accounts for ~75% of dementia cases (ADI World Alzheimer Report, 2014; Korvatska et al., 2015). The rapid increase of AD prevalence is a challenge to the public health and causes a huge socioeconomic burden worldwide. However, no effective treatment has been developed. Progressive memory loss is often the earliest sign of AD, while the impairment of other cognitive functions and psychosis are also presented (Hort et al., 2010; McKhann et al., 2011; Segal-Gidan et al., 2011).

Early-onset AD (EOAD) and late-onset AD (LOAD), occurring before and after the age of 65 years, respectively, are the two types of AD. Less than 5% of AD cases are EOAD (Alzheimer’s Association, 2016). EOAD is caused by genetic alterations, including pathogenic mutations in the amyloid-β precursor protein (*APP*) gene (Goldgaber et al., 1987; Kang et al., 1987; Robakis et al., 1987; St. George-Hyslop et al., 1987; Tanzi et al., 1987), presenilin 1 (*PSEN1*) gene (Mullan et al., 1992; Schellenberg et al., 1992; St. George-Hyslop et al., 1992; Li et al., 1995; Sherrington et al., 1995) and presenilin 2 (*PSEN2*) gene (Levy-Lahad et al., 1995a,b; Rogaev et al., 1995), a duplication of *APP* locus, as well as trisomy of chromosome 21 causing Down syndrome (DS; Campion et al., 1999; Bettens et al., 2013). The etiology of LOAD is not yet fully understood. A combination of multiple factors is believed to contribute to the pathogenesis of LOAD, including aging, genetics, nutrition, lifestyle and chronic metabolic disorders (ADI; Qiu et al., 2009; Yang and Song, 2013; Kang et al., 2017; Zeng et al., 2017; Zhang and Song, 2017). Among them, aging has been demonstrated as the greatest risk factor of AD. Due to the rapid increase in global aging population, the AD prevalence will be continuously increased worldwide (ADI; Korvatska et al., 2015).

Both EOAD and LOAD share the same pathological hallmarks in the brain, including extraneuronal neuritic plaques, intraneuronal neurofibrillary tangles and synaptic/neuronal loss leading to brain atrophy. As one of the major characteristics of AD neuropathology, neuritic plaque is mainly composed of amyloid β (Aβ), which was first identified by Glenner and Wong (1984). Thus, it has been proposed that Aβ overloading and plaque formation initiate the cascade of AD pathogenesis and also contribute to other pathological features, such as neurofibrillary tangles and synaptic/neuronal loss (Hardy and Higgins, 1992). Recent studies suggested that soluble Aβ oligomers might be the main culprit of neuron toxicity. Thus, amyloid hypothesis has been revised to propose that Aβ oligomers play a more important role in AD pathogenesis than mature amyloid fibrils do, indicating that reducing Aβ generation, facilitating Aβ clearance and blocking Aβ oligomerization would be potential strategies to inhibit the pathogenesis of AD (Sun et al., 2006a; Walsh and Selkoe, 2007; Qing et al., 2008; Karran et al., 2011; Ly et al., 2013; Dong et al., 2015).

Aβ is derived from sequential cleavages of the amyloid precursor protein (APP) by β- and γ-secretase. Over 30 pathogenic mutations in APP have been identified to cause early-onset familial AD due to the dysregulation of Aβ generation (Deng et al., 2013; Zhang S. et al., 2017). Overexpression of APP results in the elevation of Aβ levels, which is also implicated in AD pathogenesis (Brouwers et al., 2006; Rovelet-Lecrux et al., 2006, 2007; Sleegers et al., 2006; Ryoo et al., 2007; Kasuga et al., 2009; Sun et al., 2011, 2014; Long et al., 2012; Wu and Song, 2013; Yang et al., 2013; Wu et al., 2014, 2015; Song et al., 2015). For example, rare cases with *APP* locus duplication develop EOAD (Rovelet-Lecrux et al., 2006, 2007; Sleegers et al., 2006; Kasuga et al., 2009). In addition, DS patients with an extra copy of *APP* gene show the increase of APP expression and Aβ generation in the brain, which is associated with the development of AD neuropathology (Ryoo et al., 2007; Sun et al., 2011, 2014; Wu and Song, 2013; Wu et al., 2014, 2015; Song et al., 2015). Moreover, AD-associated mutations within *APP* gene promoter region also enhance APP expression (Brouwers et al., 2006). The downregulation of MiR-106b or MiR-153, targeting *APP* mRNA, has been observed in patients of sporadic AD with the elevation of *APP* mRNA (Long et al., 2012). More importantly, emerging evidence shows that alterations of APP trafficking and post-translational modifications have significant effects on APP processing and Aβ production. Therefore, we aim to introduce the current knowledge of APP modifications and trafficking, review their important roles in APP processing and Aβ generation, and discuss the effect of aberrant post-translational modifications and trafficking on Aβ generation, which may provide novel strategies for drug development in AD.

## APP Gene and Protein

The human *APP* gene is located on chromosome 21q21.3, spanning approximately 290,586bp of genomic DNA (Goldgaber et al., 1987; Kang et al., 1987; Robakis et al., 1987; Tanzi et al., 1987; Yoshikai et al., 1990; Lamb et al., 1993). By alternative splicing, approximate ten *APP* variants are generated, encoding APP isoforms with 639–770 amino acids. The three major isoforms are APP695, APP751 and APP770, all of which can generate Aβ after sequential cleavages by β- and γ- secretase (Neve et al., 1988; Tanzi et al., 1988; Zimmermann et al., 1988; Kang and Müller-Hill, 1990; Sisodia et al., 1993).

APP is ubiquitously expressed in human tissues with high expression in the central nervous system (CNS). Both APP751 and APP770 isoforms are mainly expressed in non-neuronal cells, while APP695 isoform is predominantly expressed in neurons. As the major isoform in human brains, APP695 expression is markedly increased during neuronal differentiation (Kang and Müller-Hill, 1990; Sisodia et al., 1993). The three isoforms share the conserved protein structure with a larger extracellular domain, a short transmembrane domain and a cytoplasmic domain (Muresan and Ladescu Muresan, 2015). The large ectodomain includes a cysteine-rich globular domain (E1), an acidic domain (AC), a helix-rich domain (E2) and a part of the Aβ domain extending into the transmembrane domain. The short cytoplasmic domain (the intracellular C-terminal domain) contains a conserved YENPTY motif responsible for the protein interactions. E1 domain contains a heparin-binding site (HBD) and a metal-binding domain (MBD) with copper and zinc binding sites. The E2 domain is composed of six α-helices forming a coiled-coil substructure. Both APP770 and APP751 contain a Kunitz-type serine protease inhibitors (KPI) domain following the AC. In addition, APP770 contains an OX2 domain following the KPI domain. APP plays numerous functions, such as neuronal differentiation, neurogenesis, synaptic function, apoptosis and cell proliferation (Bolós et al., 2014; Milosch et al., 2014; Fanutza et al., 2015; Wu et al., 2015, 2016).

## APP Processing and Aβ Generation

### Overview of APP Processing and Aβ Generation

**Figure 1:**
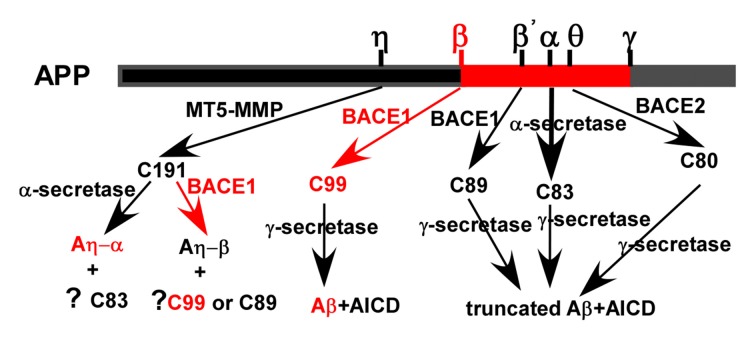
Amyloid precursor protein (APP) processing and amyloid β (Aβ) generation. APP is mainly cleaved by α-secretase to generate secreted APP (sAPPα) and C-terminal fragment (CTF) of 83 amino acids (C83). C83 is further cleaved by γ-secretases to generates a truncated Aβ and APP intracellular domain (AICD), respectively. The minority of APP is cleaved by beta-site APP cleaving enzyme 1 (BACE1; β-secretase) at Asp1 and Glu11 (numbering for Aβ) sites to generate a CTF with 99 and 89 amino acids (C99 and C89), respectively. They were further cleaved by γ-secretase to produce Aβ and a truncated form of Aβ, respectively. APP is proteolyzed by BACE2 (θ-secretase) to generate a CTF with 80 amino acids (C80), which is further cleaved by γ-secretase to produce a truncated form of Aβ. Membrane type 5 matrix metalloproteinase (MT5-MMP; η-secretase) is revealed to cleave APP generating C191, which is further cleaved by α-secretase and β-secretase to produce Aη-α and Aη-β, respectively. However, the generation of C83, C99, C89 and their downstream cleavage products following η- cleavage remains elusive.

Although Aβ is a well-known proteolytic product of APP, APP indeed undergoes both non-amyloidogenic and amyloidogenic pathways mediated by sequential cleavages of α-/β-/θ-/η-secretase and γ-secretase (Figure 1). The majority of APP undergoes non-amyloidogenic pathway. First, APP is cleaved by α-secretase to generate a N-terminal secreted APP (sAPPα) and C-terminal fragment (CTF) of 83 amino acids (C83) which is further cleaved by γ-secretase to release a 3 kDa product (P3) and APP intracellular domain (AICD). In addition, beta-site APP cleaving enzyme 2 (BACE2) is a θ-secretase, which is implicated in APP processing without Aβ generation (Sun et al., 2006b; Liu et al., 2013). The minority of APP is cleaved by β-secretase at Asp1 (β site) and Glu11 (β’ site, numbering for Aβ) sites, respectively. Glu11 is the major β-cleavage site to yield a CTF with 89 amino acids (C89), which is further cleaved by γ-secretase to produce a truncated Aβ_11-40/42._ Asp1 is the minor β-cleavage site to generate a CTF with 99 amino acids (C99; Deng et al., 2013). C99 is further cleaved by γ-secretase to produce Aβ (Liu et al., 2002; Deng et al., 2013). Recently, η-secretase, e.g., membrane type 5 matrix metalloproteinase (MT5-MMP), is revealed to be involved in APP processing (Willem et al., 2015).

### α-Secretase

Although α-secretase is not yet fully defined, three a disintegrin and metalloproteinase (ADAM) family members (ADAM9, ADAM10 and ADAM17) may feature α-secretase activity. α-cleavage predominantly occurs at plasma membrane (PM) to generate sAPPα and C83 excluding Aβ production, which is the major proteolytic process of APP at PM (Sisodia, 1992). The activity of α-secretase in Trans-Golgi-Network (TGN) v is regulated by multiple factors such as protein kinase C (PKC; Skovronsky et al., 2000).

### Beta-Site APP Cleaving Enzyme 1

BACE1, the dominant β-secretase *in vivo*, is a type-I transmembrane protein with 501 amino acids (Sun et al., 2012). The trafficking of BACE1 has been reviewed previously (Zhang and Song, 2013; Agostinho et al., 2015; Toh and Gleeson, 2016). Briefly, it is synthesized in the endoplasmic reticulum (ER)-bound polysomes as immature BACE1. Along the secretory pathway, BACE1 undergoes a series of post-translational- modifications in the ER and Golgi apparatus, becoming mature. Mature BACE1 is internalized through endosomes to lysosomes for degradation. It is also transported to the TGN via the retrograde route or recycled to the PM. BACE1 mainly localizes in the TGN and endosome where the acidic environment is optimal for BACE1 activity (Vassar et al., 1999). Thus, β-cleavage mainly occurs in the post-Golgi secretory compartments and endosomal/lysosome organelles (Koo and Squazzo, 1994; Haass et al., 1995; Munger et al., 1995). However, accumulated evidence has shown that intracellular β-secretase cleavage also occurs in ER/ER-Golgi Intermediate Compartment (ERGIC), indicating that acidic pH is not essential for BACE1 activity (Chyung et al., 1997). More importantly, the site preference of β-cleavage is mainly determined by the subcellular location and modification of BACE1 (Huse et al., 2002; Wang et al., 2014). Asp1 is the major BACE1 cleavage site in the ER resulting in C99 generation, which contributes to Aβ generation. However, Glu11 is the predominant β-cleavage site in the TGN, leading to C89 generation without the subsequent Aβ production. In addition, lipid raft is the preferred microdomain for β-secretase cleavage (Zhang and Song, 2013). Many BACE1 inhibitors have been developed for AD treatment by inhibiting the generation of Aβ (Godyń et al., 2016; Hung and Fu, 2017). However, none of them is approved so far.

### θ-Secretase

BACE2, the homolog of BACE1, consists of 518 amino acids (Sun et al., 2005). BACE2 is a θ-secretase, which predominantly cleaves APP at Phe19 within the Aβ domain to yield CTF of 80 amino acids (C80) excluding Aβ generation (Figure 1; Sun et al., 2006b; Liu et al., 2013). Consistently, no Aβ overproduction and cognitive deficits were observed in BACE2 transgenic mice (Azkona et al., 2010; Bacher et al., 2010). Despite of high homology, the expression of BACE2 and BACE1 is differentially regulated at transcriptional and post-transcriptional levels (Sun et al., 2005, 2006b). BACE1 is predominantly expressed in neurons, whereas BACE2 expression is extremely low in the brain (Bennett et al., 2000; Marcinkiewicz and Seidah, 2000).

### γ-Secretase

γ-secretase is a protein complex consisting of presenilins (PSEN1 and PSEN2), nicastrin, APH-1 and PEN-2, which cleaves APP following α-/β-/θ-secretase cleavage. PSEN1 and PSEN2 are the core catalytic subunits of γ-secretase, while nicastrin, APH-1 and PEN-2 are the regulatory subunits playing a key role in the maturation and stabilization of the complex (De Strooper et al., 1998, 1999; Song et al., 1999; Zhang et al., 2000, 2013; Takasugi et al., 2003). In addition, Chen et al. (2006) showed that TMP21, a vesicle trafficking protein, is a component of γ-secretase, and its dysregulation and SNPs significantly affect Aβ generation (Chen et al., 2006; Zhang X. et al., 2017). Studies suggest that all the components of the complex are synthesized and mainly localized in the ER (Walter et al., 1996). Most evidence supports that the assembly and maturation processes, mainly occurring in the compartments of the secretory pathway, are crucial for γ-secretase activity, although one report showed that presenilins are not required for Aβ generation in the early secretory pathway (Wilson et al., 2002; Zhao et al., 2004; Capell et al., 2005). The details of γ-secretase trafficking and assembly have been extensively reviewed (Zhang et al., 2013; Agostinho et al., 2015). The active γ-secretase is mainly localized in late endosome and lysosome system (Kanatsu et al., 2014). However, the γ-cleavage has also been observed in the ER, TGN and at PM (Munger et al., 1995; Walter et al., 1996; Maltese et al., 2001; Zhao et al., 2004; Capell et al., 2005; Kaether et al., 2006). Targeting γ-cleavage is a well-known strategy for AD treatment by inhibiting Aβ generation. Although many γ-cleavage inhibitors have been developed, none of them is approved for clinical application (Godyń et al., 2016; Hung and Fu, 2017).

### η-Secretase

Membrane type 1 matrix metalloproteinase (MT1-MMP) was first revealed to be involved in APP processing (Higashi and Miyazaki, 2003). Later on, Ahmad et al. (2006) found that MT3-MMP and MT5-MMP also contribute to APP processing. However, MT3-MMP has no effect on Aβ generation (Ahmad et al., 2006). Recently, Willem et al. (2015) demonstrated that MT5-MMP has η-secretase activity, which cleaves APP695 at amino acids 504–505 to generate a higher molecular mass carboxy-terminal fragment of APP, termed CTF-η (C191). C191 is enriched in an AD mouse model and human AD brains and it could be further processed by α- and β-secretase generating Aη-α and Aη-β, respectively (Figure 1; Willem et al., 2015). Aη-α significantly inhibits long-term potentiation *in vitro* and *in vivo* (Willem et al., 2015). However, the generation of C83, C99, C89 and downstream cleavage products following η-cleavage remains elusive although Baranger et al. (2016, 2017) reported that MT5-MMP is a pro-amyloidogenic secretase, promoting amyloid pathology and cognitive decline in AD model mice.

### Aβ Generation Along the Secretory Pathway

Aβ, the major component of senile plaques in AD brains, is generated through sequential cleavages of APP by β- and γ-secretase. The trafficking-dependent co-residence of APP and secreatases plays a key role in the Aβ generation. However, the subcellular location of Aβ production is not fully defined. Several studies have shown that Aβ is mainly generated in the endosome/lysosome where the acidic environment facilitates the activity of β- and γ-secretase (Golde et al., 1992; Koo et al., 1996; Yamazaki et al., 1996; Kanatsu et al., 2014). In contrast, a large body of evidence indicates that Aβ is also generated along the secretory pathway, including ER, Golgi and TGN (Busciglio et al., 1993; Stephens and Austen, 1996; Cook et al., 1997; Hartmann et al., 1997; Tomita et al., 1998; Greenfield et al., 1999; McFarlane et al., 1999). It has been found that Aβ42 instead of Aβ40 is generated in the ER of neurons (Cook et al., 1997; Hartmann et al., 1997; Greenfield et al., 1999). Consistently, ER retention signal or BFA treatment-induced ER retention of APP increases the level of Aβ42. Since both α- and β-secretases exert their cleavages in the TGN, there is a competition between α-secretase and β-secretase for APP cleavage (Skovronsky et al., 2000).

## APP Modifications and Trafficking in Aβ Generation

### APP Trafficking and Co-Residence with Secretases

**Figure 2:**
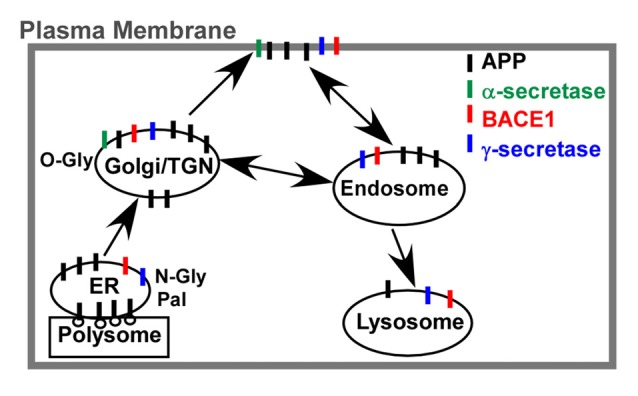
APP trafficking and co-residence with secretases. After synthesized in the membrane-bound polysomes, the N-terminal signal peptide is removed during its translocation into the endoplasmic reticulum (ER). N-glycosylation (N-Gly) and palmitoylation (Pal) is crucial for APP transporting to the Golgi apparatus and Trans-Golgi-Network (TGN), while O-glycosylation (O-Gly) in Golgi apparatus is essential for APP transporting to the plasma membrane (PM). The uncleaved APP is rapidly internalized by the endosome and sorted into three pathways. Most APP is sorted into late endosome-lysosome pathway for degradation while a small fraction of APP is recycled back to the PM or retrograded to the TGN. Moreover, the co-residence of APP with secretases (e.g., α-, β-, γ-secretase) along the secretory pathway might contribute to the APP processing and Aβ generation.

As a type I transmembrane protein, APP trafficks through the classic secretory, endo-lysosome and recycling pathways (Figure 2). After synthesized in the membrane-bound polysomes, the N-terminal signal peptide is removed during its translocation into the ER. Then, it transports to the Golgi apparatus and TGN via ERGIC. Finally, approximately 10% nascent APP reaches the PM, while the majority of APP resides in the Golgi apparatus and TGN. APP at PM is mainly cleaved by α-secretase to release sAPP and C83. The uncleaved APP on the cell surface is rapidly internalized into the endosome, which is mediated by its C-terminal “YENPTY” motif. The internalized APP is sorted into three pathways. Most APP is sorted into late endosome-lysosome pathway for degradation while a small fraction of APP is recycled back to the cell surface or retrograded to the TGN (Haass et al., 1992). Moreover, part of APP at TGN could be directly sorted into the endosome. APP and its fragments have also been detected in the mitochondria, cytoplasm and nuclear. However, the detailed routes and processes remain elusive since the holo-APP and multiple APP fragments may traffic through different pathways (Muresan and Ladescu Muresan, 2015). Furthermore, the trafficking of APP plays a key role in APP processing as the co-residence of APP with secretases (e.g., α-, β-, γ-secretase) along the secretory pathway and organelle-specific secretase activity significantly affect APP processing and Aβ generation (Figure 2; Zhang and Song, 2013; Zhang et al., 2013; Agostinho et al., 2015). For example, α-cleavage predominantly occurs at PM (Sisodia, 1992), while β-cleavage mainly occurs in the endosome and lysosome (Koo and Squazzo, 1994; Haass et al., 1995; Munger et al., 1995).

### Post-Translational Modifications of APP

During the constitutive secretory pathway, APP undergoes extensive post-translational modifications, including N-glycosylation (N-Gly) and O-glycosylation (O-Gly), phosphorylation, sulfation, palmitoylation, ubiquitination and sumoylation. The residue numbering in the following corresponds to the APP695, unless otherwise indicated.

#### Glycosylation

When the nascent APP translocates into the ER, N-Gly is catalyzed by the oligosaccharyl transferase (OST) complex with the addition of a precursor oligosaccharide to the luminal side of a polypeptide chain, forming the immature APP. Two asparagine sites, Asn467 and Asn496, are predicted to be glycosylated although only the former one has been confirmed (Pahlsson et al., 1992). However, Yazaki et al. (1996) showed that deletion of either Asn467 or Asn496 leads to a decrease of APP molecular weight in COS-1 cells, which indirectly indicates that both sites are N-glycosylated. O-Gly of APP occurs in Golgi apparatus to form the mature APP. Multiple O-Gly sites of APP have been identified by both *in vitro* and *in vivo* studies. Thr291, Thr292, Thr576 and Thr353 (numbering of APP770) are found to be O-Glycosylated in cultures (Perdivara et al., 2009). The O-Gly of Ser597, Ser606, Ser611, Thr616, Thr634, Thr635, Ser662 and Ser680 (numbering of APP770) has also been identified in human CSF (Halim et al., 2011). In addition to the classical O-GalNAcylation, O-GlcNAcylation is another form of O-Gly and characterized by the addition of a single β-N-acetylglucosamine (GlcNAc) to the residue of serine or threonine (Griffith et al., 1995). Alteration of O-GlcNAcylation in APP plays an important role in regulating APP processing and Aβ generation (Jacobsen and Iverfeldt, 2011; Chun et al., 2015b).

#### Phosphorylation

Although APP is a phosphoprotein, the fully glycosylated (N-and O-glycosylated) APP is preferred to be phosphorylated (Gandy et al., 1988; Weidemann et al., 1989; Hung and Selkoe, 1994; Oishi et al., 1997). Ten phosphorylated sites of APP have been identified, including two sites in the ectodomain (Ser198 and Ser206) and eight sites in the cytoplasmic domain (Tyr653, Tyr682, Tyr687, Ser655, Ser675, Thr654, Thr668 and Thr686; Gandy et al., 1988; Walter et al., 1997; Lee et al., 2003). Under basal conditions, two phosphorylated serine residues (Ser198 and Ser206) could be detected in the ectodomain of APP and Ser198 is the major phosphorylated site compared with Ser206 (Hung and Selkoe, 1994; Walter et al., 1997).

More studies about APP phosphorylation focus on the residues within the cytoplasmic domain. Ser655 can be phosphorylated by PKC, while Ca^++^/calmodulin-dependent protein kinase II is involved in the phosphorylation of both Ser655 and Thr654 during *in vitro* culture (Gandy et al., 1988; Suzuki et al., 1992). Moreover, Ser655 can be phosphorylated by APP kinase I *in vivo* (Isohara et al., 1999). Oishi et al. (1997) has reported that okadaic acid, a protein phosphatase 1 (PP1) and PP2A inhibitor, increases Ser655 phosphorylation, suggesting that phosphatases are also involved in the regulation of Ser655 phosphorylation. Phosphorylation of Ser655 is mainly detected in the mature APP, whereas Thr668 is the most common phosphorylated site in the immature APP (Oishi et al., 1997). The phosphorylation of Thr668 occurs in the ER and is cell-cycle dependent (Muresan and Muresan, 2012). Multiple kinases, such as glycogen synthase kinase 3β (GSK3β), cyclin dependent kinase 5 (CDK5), CDK1, stress-activated protein kinase1β (SAPK1β), dual-specificity tyrosinephosphorylation-regulated kinase 1A (DYRK1A) and c-Jun N-terminal protein kinase (JNK) are involved in the process of its phosphorylation (Suzuki et al., 1994; Aplin et al., 1996; Oishi et al., 1997; Iijima et al., 2000; Standen et al., 2001; Ryoo et al., 2008; Mazzitelli et al., 2011). In addition, a couple of phosphatases, such as PP1, PP2A, PP2B are also involved in the regulation of Thr668 phosphorylation (Oliveira et al., 2015).

#### Palmitoylation

Palmitoylation is a common way of protein modifications with the addition of fatty acids to a cysteine residue, which is regulated by both palmitoyl acyltransferases and acyl protein thioesterases. Protein palmitoylation is involved in the regulation of protein trafficking and protein-protein interactions. Around 10% of APP undergoes palmitoylation, mainly occurring in ER (Bhattacharyya et al., 2013). Recently, Bhattacharyya et al. (2013) has reported that APP palmitoylation is mediated by two palmitoyl acyltransferases, DHHC-7 and DHHC-21, and Cys186 and Cys187 are two palmitoylated sites in APP.

#### Ubiquitination

Ubiquitination can modify the target protein by attaching ubiquitin, a small protein with 76 amino acids, to the lysine residues. It is catalyzed by ubiquitin-activating enzymes, ubiquitin-conjugating enzymes and ubiquitin ligases. Protein ubiquitination is implicated in the processes including protein degradation, trafficking and protein-protein interactions. Recent studies have identified a couple of ubiquitination sites within the cytoplasmic domain of APP, including Lys649–651, Lys651 and Lys688 (Kaneko et al., 2010; El Ayadi et al., 2012; Watanabe et al., 2012; Morel et al., 2013).

#### Sumoylation

Protein sumoylation is characterized by the covalent modification of lysine residues on target proteins with small ubiquitin-like modifier (SUMO; SUMO-1, -2 and -3). It is an important modification to regulate protein functions and catalyzed by SUMO E1, E2 and E3 enzymes. It has been identified that both SUMO-1 and -2 are implicated in APP sumoylation with two sumoylated sites, Lys587 and Lys595 (Zhang and Sarge, 2008).

#### Sulfation

Tyrosine sulfation is a common post-translational modification of cell surface protein occurring in the late Golgi compartments, which is implicated in protein trafficking as well as proteolysis process. APP is a tyrosine sulfated protein with two potential sulfated residues, Tyr217 and Tyr262 (Weidemann et al., 1989). However, the exact sulfated sites and the function of APP sulfation have not been fully investigated.

#### Interplay of Post-Translational Modifications

Growing evidence indicates that the interplay of post-translational modifications, including phosphorylation and O-GlcNAcylation, phosphorylation and sumoylation, sumoylation and ubiquitination, and O-GlcNAcylation and sumoylation, is involved in complex physiological processes and the pathogenesis of multiple diseases (Zeidan and Hart, 2010; Hart et al., 2011; Ruan et al., 2013; Luo et al., 2014; Liebelt and Vertegaal, 2016). For example, alternative phosphorylation and O-GlcNAcylation of insulin receptors substrates is implicated in the risk of AD and diabetes (Jahangir et al., 2014). In addition, sumoylation of Tau protein promotes Tau phosphorylation and inhibits its ubiquitination, contributing to AD-associated Tau hyperphosphorylation and accumulation (Luo et al., 2014). As phosphorylation, O-GlcNAcylation, sumoylation and ubiquitination are all involved in APP modification, the interplay of APP modifications may affect APP processing and Aβ generation. For example, both phosphorylation and O-GlcNAcylation occur on serine and threonine residues, suggesting that these two types of post-translational modifications may have reciprocal effects at the same site contributing to the regulation of APP processing and Aβ generation (Weidemann et al., 1989; Chou et al., 1995; Cheng and Hart, 2001; Zeidan and Hart, 2010). However, the interplay of APP modifications has not been reported so far. Therefore, further investigation is essential to elucidate the interplay among APP modifications and its role in AD pathogenesis, providing a novel insight into AD treatment.

### Modifications and Trafficking

Along the secretory pathway, APP is subject to post-translational modifications. On the other hand, APP modifications affect its sorting and trafficking (Table 1). Thus, APP modifications and trafficking are mutually regulated, contributing to the regulation of Aβ generation.

**Table 1:** Amyloid precursor protein (APP) modifications and trafficking in amyloid β (Aβ) generation.

Modification	Trafficking	Affected cleavage	Aβ	Cells/Organs	References
Glycosylation
N-Glycosylation				COS-7 HEK293	Pahlsson et al. (1992)
O-Glycosylation	PM	α-secretase	↓	PC12 CHO Hela	Weidemann et al. (1989) Perdivara et al. (2009) Jacobsen and Iverfeldt (2011) Chun et al. (2015a,b)
Palmitoylation
Cys186/187	Lipid raft	β-secretase	↑	CHO PC12 Primary neuron	Bhattacharyya et al. (2013)
Phosphorylation
Ser655	TGN	α-secretase	↓	PC12 Rat brains	Gandy et al. (1988) Buxbaum et al. (1990) Suzuki et al. (1992) Isohara et al. (1999)
Thr668		β-/γ-secretase	↑	SKSH-SY5Y H4 AD brains Mouse brains Primary neuron	Lee et al. (2003) Vingtdeux et al. (2005) Judge et al. (2011) Mazzitelli et al. (2011)
Tyr687	ER/Golgi	α-/γ-secretase	↓	AD brains COS-7 HEK293	Zambrano et al. (2001) Tarr et al. (2002) Lee et al. (2003) Rebelo et al. (2007) Takahashi et al. (2008)
Ubiquitination
Lys651	PM ↓ Lipid raft	α-secretase ↓β-secretase	↓ ↓	Primary neuron Hela HEK293 N2a Mouse brains	Watanabe et al. (2012) Morel et al. (2013)
Lys688	Golgi		↓	PC12, H4 and HEK293	Hiltunen et al. (2006) El Ayadi et al. (2012)
Sumoylation
Lys587/Lys595			↓	Hela	Zhang and Sarge (2008)
Sulfation				PC12	Weidemann et al. (1989)

#### Glycosylation and Trafficking

N-Gly and O-Gly primarily occurs in the ER and Golgi/TGN, respectively. Both of them are essential for APP trafficking (Greenfield et al., 1999). The N-glycosylated APP (i.e., immature APP) is mainly located in the ER, while the mature APP generated through N-Gly, O-Gly and other modifications (e.g., sulfation, palmitoylation and phosphorylation) is mainly located in the TGN and at the PM (Tomita et al., 1998). Weidemann et al. (1989) reported that only the mature form of APP is detected on the cell surface, indicating that both N- and O-Gly are required for APP trafficking to the PM. A study by Chun et al. (2015b) has also shown that O-GlcNAcylation facilitates APP trafficking from the TGN to the PM, but inhibits the endocytosis of APP from the PM. Moreover, the sorting of APP from the Golgi apparatus to the cell surface is prevented due to the deletion of two N-glycosylated sites, Asn467 and Asn496 (Yazaki et al., 1996; McFarlane et al., 1999). Blockade of N-Gly can also inhibit the transport of APP to axonal synaptic membrane (McFarlane et al., 2000). Aforementioned evidence indicates that glycosylation plays a pivotal role in APP trafficking.

#### Phosphorylation and Trafficking

APP phosphorylation occurs along the secretory pathway and modulates its sorting and trafficking (Knops et al., 1993; Walter et al., 1997). For example, a APP mutant mimicking the constitutive phosphorylated Tyr687 retains APP in the ER and Golgi. In contrast, a dephosphomimetic APP mutant, the whose phosphorylation site Tyr687 was substituted by alanine, markedly reduces the expression of APP on the cell surface (Rebelo et al., 2007; Takahashi et al., 2008). Phosphorylation of Ser655 also potentiates APP sorting and trafficking from the endosome to the TGN, but attenuates its trafficking to the lysosomes (Vieira et al., 2010). Accordingly, the dephosphomimetic Ser655Ala mutant is preferred to be targeted for lysosomal degradation (Vieira et al., 2010).

#### Palmitoylation and Trafficking

APP palmitoylation is essential for APP trafficking and maturation. Double mutations at two palmitoylation sites, Cys186 and Cys187, result in the ER retention of APP and the blockade of APP maturation. Moreover, palmitoylated APP is highly enriched in lipid rafts (Bhattacharyya et al., 2013).

#### Ubiquitination and Trafficking

Ubiquitination of APP also affects its sorting and trafficking. Abolishing ubiquitination by substituting Lys649–651 with arginines inhibits the sorting of APP into endosomal intraluminal vesicles (ILVs) in both Hela cells and hippocampal neurons (Morel et al., 2013). In addition, K63-linked polyubiquitination of APP inhibits APP maturation and impairs APP trafficking by sequestering it in the early secretory pathway, such as the Golgi apparatus. The substitution of Lys688 with arginine dramatically reduces APP ubiquitination and Golgi sequestration (El Ayadi et al., 2012). Moreover, FBL2-induced APP ubiquitination inhibits APP endocytosis resulting in an increase of APP on the cell surface and a decrease of APP in lipid rafts (Watanabe et al., 2012).

### Aberrant APP Modifications and Trafficking Dysregulate Aβ Generation in AD

Aberrant APP modifications and impairment of APP trafficking have been found in AD patients (Lee et al., 2003; Placido et al., 2014; Joshi and Wang, 2015), which plays an important role in the regulation of APP processing and Aβ generation (Table 1). Compared with the impairment of APP trafficking, the role of aberrant APP modifications in AD pathogenesis have been studied more extensively. Therefore, the dysregulation of trafficking is incorporated into the alteration of modifications.

#### Phosphorylation and Aβ Generation

Dysregulation of multiple kinases and phosphatases has been observed in AD brains, including GSK3, PKC, DYRK1A, PP1, PP2A (Wang et al., 1994; Pei et al., 1999; Ferrer et al., 2005; Braithwaite et al., 2012). The abnormality of SET and RCAN1, two phosphatase regulators, has also been found in AD and DS brains, which possibly contributes to the dysregulation of phosphatase activity (Tanimukai et al., 2005; Wu and Song, 2013; Wu et al., 2015; Zhang et al., 2015). As the substrate of above kinases and phosphatases, APP phosphorylation is impaired in AD, leading to the aberrant APP processing and Aβ generation.

The phosphorylation status of APP differentially affects APP processing and Aβ generation. Previous studies have suggested that reduction of Ser655 and ectodomain phosphorylation may stimulate Aβ generation in AD. For example, a deficiency of PKC in AD brains may reduce the phosphorylation of Ser655 and ectodomain (Wang et al., 1994), promoting Aβ generation (Buxbaum et al., 1990), while PKC-induced increased α-secretase cleavage in the TGN results in the reduction of β-cleavage and Aβ generation (Skovronsky et al., 2000). Moreover, PP1 and PP2A inhibitors have the same effect as PKC activation, such as increasing the secretion of soluble APP and reducing Aβ generation (Buxbaum et al., 1990, 1993; Hung et al., 1993; Hung and Selkoe, 1994).

Increased Thr668 phosphorylation has been detected in AD and DS brains, which is resulted from the imbalance between kinases and phosphatases. For example, increased DYRK1A in DS and AD promotes Thr668 phosphorylation (Ferrer et al., 2005; Ryoo et al., 2008; Wegiel et al., 2008). A number of studies have identified that Thr668 phosphorylation increases Aβ generation both *in vitro* and *in vivo* (Lee et al., 2003; Vingtdeux et al., 2005; Judge et al., 2011; Mazzitelli et al., 2011). Although phosphorylation of Thr668 residue reduces APP secretion, it facilitates β- and γ-secretase cleavages in neurons (Ando et al., 2001; Lee et al., 2003; Vingtdeux et al., 2005; Ryoo et al., 2008; Mazzitelli et al., 2011; Kim et al., 2016; Triaca et al., 2016), leading to enhanced generation of C99 sand Aβ (Suzuki et al., 1994; Colombo et al., 2009; Mazzitelli et al., 2011). However, two studies have reported that Thr668 phosphorylation reduces Aβ generation by inhibiting γ-secretase cleavage (Feyt et al., 2007; Matsushima et al., 2012). It has to be noted that Thr668 phosphorylation inhibits γ-secretase cleavage in CHO cells, which is different from that in neurons Although Thr668E could mimic Thr668 phosphorylation, it may have differential effects on APP conformation change compared with phosphorylated Thr668. Moreover, kinase activation and inhibition may also directly modulate the activity of α-, β- and γ-secretases in addition to APP phosphorylation.

Phosphorylated Tyr682 and Tyr687 have been detected in AD brains but not in healthy controls (Zambrano et al., 2001; Tarr et al., 2002; Lee et al., 2003; Rebelo et al., 2007). Compared with Tyr682, Tyr687 is the major tyrosine phosphorylation site (Takahashi et al., 2008). However, Tyr682 and Tyr687 phosphorylation cannot be detected in the cell lines overexpressing APP, suggesting that phosphorylation of these two residues may be exclusive in AD brains. Intriguingly, a couple of studies suggested that the phosphorylation of Tyr682 and Tyr687 negatively regulates APP processing and Aβ generation. For example, Trk A phosphorylates APP at Tyr682 and reduces the level of AICD (Tarr et al., 2002). In addition, APP mutant which mimics constitutive Tyr687 phosphorylation increases its half-life, but reduces Aβ generation in COS cells (Rebelo et al., 2007). However, a APP mutant with constitutive dephosphorylated Tyr687 (tyrosine replaced by alanine) also reduces both α- and γ-cleavages on APP (Takahashi et al., 2008). Since the cell type and conformation change of APP mutants may differently affect APP processing and Aβ generation, the role of Tyr682 and Tyr687 phosphorylation in Aβ generation remains elusive, which needs to be further investigated.

#### Glycosylation and Aβ Generation

Reduced O-GlcNAcylation is observed in AD brains (Liu et al., 2009). Recent studies showed that APP O-GlcNAcylation plays a key role in APP processing. Jacobsen and Iverfeldt (2011) reported that increased O-GlcNAcylated APP by O-GlcNAcase (OGN) inhibitor or siRNA stimulated α-cleavage of APP and reduced Aβ generation. Consistently, Chun et al. (2015b) showed that the OGN inhibitor increased α-cleavage of APP and inhibited the β-cleavage of APP. These effects are due to the inhibition of APP endocytosis, which further increases the level of APP at PM (Sisodia, 1992). In addition, O-GlcNAcylation status of α-, β- and γ-secretases may also be implicated in OGN inhibitor-induced alteration of APP processing (Dias et al., 2009; Tarrant et al., 2012). Recently, Chun et al. (2015a) showed that the APP O-glycosylated site mutant (Thr576Ala) reduced APP expression on cell surface but increased its accumulation in the early endosome, leading to the increase of Aβ generation. It indicates that APP glycosylation does affect its processing and Aβ generation, which is associated with the alteration of APP trafficking.

#### Palmitoylation and Aβ Generation

Emerging evidence indicates that APP palmitoylation plays an important role in Aβ generation. Abolishing APP palmitoylation by site-direct mutagenesis inhibits APP cleavage by α- and β-secretase, resulting in the dramatic reduction of APP-CTFs. On the hand, APP palmitoylation preferentially targets APP into the lipid rafts where BACE1 is enriched, facilitating β-cleavage by BACE1 and Aβ generation (Bhattacharyya et al., 2013).

#### Ubiquitination and Aβ Generation

It has been demonstrated that APP ubiquitination inhibits APP processing and Aβ generation. HRD1-induced APP ubiquitination facilitates APP degradation, leading to the reduction of Aβ generation (Kaneko et al., 2010). In addition, blocking APP ubiquitination by the substitution of Lys649–651with arginines impairs APP sorting and enhances Aβ generation (Morel et al., 2013), while the ubiquitination of Lys651 is essential for FBL2-induced reduction of Aβ generation (Watanabe et al., 2012). Moreover, ubiquilin 1-mediated K63-linked polyubiquitination of APP delays its proteolytic processing, while reduced UBQLN1 increases Aβ generation (Hiltunen et al., 2006; El Ayadi et al., 2012).

#### Sumoylation and Aβ Generation

Two sumoylation sites of APP, Lys587 and Lys595, are close to the β-cleavage site, suggesting that sumoylation of APP may affect β-cleavage of APP and Aβ generation. Simultaneous overexpression of Ubc9 and SUMO-1 promotes APP sumoylation, which is associated with the reduction of Aβ generation. The result indicates that APP sumoylation may negatively regulate Aβ generation (Zhang and Sarge, 2008). In addition, SUMO3 reduces APP turnover rate, which may also contribute to the alteration of Aβ generation (Dorval et al., 2007).

### Therapeutic Strategies for AD by Targeting Aβ

Currently, only four symptomatic drugs, rivastigmine, donepezil, galantamine and memantine, are available for AD treatment by regulating cholinergic and glutamatergic systems, which only leads to a temporary slowdown in the loss of cognitive function. However, these drugs neither delay the progression of dementia nor represent a cure (Godyń et al., 2016; Hung and Fu, 2017). As there is no effective treatment for AD, it is urgent to develop novel drugs for AD treatment. Targeting Aβ generation e is a major strategy for drug development in addition to accelerating Aβ clearance, anti-Tau pathology and anti-inflammation strategies (Godyń et al., 2016; Hung and Fu, 2017). Up to now, more than a hundred of BACE1 inhibitors and γ-secretase modulators have been developed for AD treatment by inhibiting the generation of Aβ. However, none of them is approved although a couple of inhibitors are still in the clinical trial (Godyń et al., 2016; Hung and Fu, 2017). The failure of many inhibitors in clinical trial suggests that several key issues need to be considered. First, as there are tons of known and unknown substrates of BACE1 and γ-secretase, the effects of the inhibitors on other substrates and associated processes should be considered. For example, the severe side effect of the first generation of γ-secretase inhibitor is caused by inhibiting Notch cleavage, a major substrate of γ-secretase (Song et al., 1999; Qing et al., 2008). Second, the inhibition effect on site preference of BACE1 and γ-secretase should be considered because only β-site and γ-site cleavages by BACE1 and γ-secretase contribute to Aβ generation but not β’-site and γ-site cleavages by BACE1 and γ-secretase, respectively (Deng et al., 2013; Zhang et al., 2013). In addition, the effect of the inhibitors on the co-residence of APP with BACE1 and γ-secretase should be considered, which is more important than the general inhibition of BACE1 and γ-secretase activity. Moreover, modulating APP modification might be a novel strategy to complement current strategies of inhibiting BACE1 and γ-secretase activity, which has two major advantages. First, it would have less or no effect on the processing of other BACE1 and γ-secretase substrates, resulting in less or no side effect. Second, it could reduce the co-residence of APP with BACE1 and γ-secretase by altering APP trafficking leading to the reduction of Aβ generation. Importantly, it may alter the cleavage site preference contributing to the reduction of Aβ generation as two recent studies reported that the APP mutants significantly affect cleavage site preference of BACE1 (Kimura et al., 2016; Zhang S. et al., 2017).

## Conclusion

**Figure 3:**
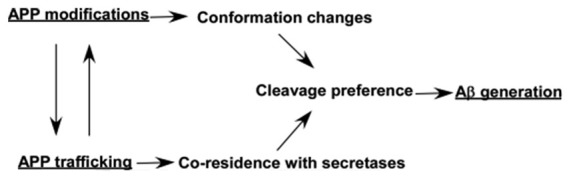
Effects of modifications and trafficking of APP on its processing and Aβ generation. Post-translational modifications of APP occur along the constitutive secretory pathway, while the modifications also affect APP trafficking. Both APP modifications-induced conformation changes and trafficking-dependent co-residence with different secretases may alter the cleavage preference of each secretase, resulting in the alteration of APP processing and Aβ generation.

Post-translational modifications of APP occur along the constitutive secretory pathway, while it also affect APP trafficking (Figure 2 and Table 1), indicating that APP modifications and trafficking are mutually regulated (Figure 3). The modifications and trafficking of APP are precisely controlled to execute its physiological functions and maintain its normal processing. A growing body of evidence has shown that modifications and trafficking of APP have significant effects on APP processing and Aβ production. Aberrant APP modifications-induced trafficking and conformation changes may alter the cleavage preference of each secretase, resulting in the dysregulation of APP processing and Aβ generation (Figure 3). Moreover, alteration of APP trafficking has significant effects on its co-residence with different secretases, contributing to the alterations of APP processing and Aβ generation (Figure 3). Thus, the regulation of APP modifications and trafficking needs to be further investigated in order to develop novel therapeutic approaches for AD by modulating APP modification and trafficking.

## Author Contributions

XW, XZ, GL, YZ and YW wrote the manuscript. YW and WS formatted and revised the manuscript.

## Conflict of Interest Statement

The authors declare that the research was conducted in the absence of any commercial or financial relationships that could be construed as a potential conflict of interest.

## References

[B1] ADI world alzheimer report (2014). ADI World Alzheimer Report, 2014. London: Alzheimer’s Disease International (ADI) Available online at: https://www.alz.co.uk/research/WorldAlzheimerReport2014.pdf

[B2] Agostinho P. Pliássova A. Oliveira C. R. Cunha R. A. (2015). Localization and trafficking of amyloid-β protein precursor and secretases: impact on Alzheimer’s disease. J. Alzheimers Dis. 45, 329–347. 10.3233/JAD-142730 25589722

[B3] Ahmad M. Takino T. Miyamori H. Yoshizaki T. Furukawa M. Sato H. (2006). Cleavage of amyloid-β precursor protein (APP) by membrane-type matrix metalloproteinases. J. Biochem. 139, 517–526. 10.1093/jb/mvj054 16567416

[B4] Alzheimer’s Association. (2016). 2016 Alzheimer’s disease facts and figures. Alzheimers Dement. 12, 459–509. 10.1016/j.jalz.2016.03.001 27570871

[B5] Ando K. Iijima K. I. Elliott J. I. Kirino Y. Suzuki T. (2001). Phosphorylation-dependent regulation of the interaction of amyloid precursor protein with Fe65 affects the production of β-amyloid. J. Biol. Chem. 276, 40353–40361. 10.1074/jbc.M104059200 11517218

[B6] Aplin A. E. Gibb G. M. Jacobsen J. S. Gallo J. M. Anderton B. H. (1996). *In vitro* phosphorylation of the cytoplasmic domain of the amyloid precursor protein by glycogen synthase kinase-3β. J. Neurochem. 67, 699–707. 10.1046/j.1471-4159.1996.67020699.x 8764598

[B7] Azkona G. Levannon D. Groner Y. Dierssen M. (2010). *In vivo* effects of APP are not exacerbated by BACE2 co-overexpression: behavioural characterization of a double transgenic mouse model. Amino Acids 39, 1571–1580. 10.1007/s00726-010-0662-8 20596738

[B8] Bacher M. Deuster O. Aljabari B. Egensperger R. Neff F. Jessen F.. (2010). The role of macrophage migration inhibitory factor in Alzheimer’s disease. Mol. Med. 16, 116–121. 10.2119/molmed.2009.00123 20200619 PMC2829616

[B9] Baranger K. Bonnet A. E. Girard S. D. Paumier J. M. García-González L. Elmanaa W.. (2017). MT5-MMP promotes Alzheimer’s pathogenesis in the frontal cortex of 5xFAD mice and APP trafficking *in vitro*. Front. Mol. Neurosci. 9:163. 10.3389/fnmol.2016.00163 28119565 PMC5223243

[B10] Baranger K. Marchalant Y. Bonnet A. E. Crouzin N. Carrete A. Paumier J. M.. (2016). MT5-MMP is a new pro-amyloidogenic proteinase that promotes amyloid pathology and cognitive decline in a transgenic mouse model of Alzheimer’s disease. Cell. Mol. Life Sci. 73, 217–236. 10.1007/s00018-015-1992-1 26202697 PMC4700096

[B11] Bennett B. D. Babu-Khan S. Loeloff R. Louis J. C. Curran E. Citron M.. (2000). Expression analysis of BACE2 in brain and peripheral tissues. J. Biol. Chem. 275, 20647–20651. 10.1074/jbc.M002688200 10749877

[B12] Bettens K. Sleegers K. Van Broeckhoven C. (2013). Genetic insights in Alzheimer’s disease. Lancet Neurol. 12, 92–104. 10.1016/S1474-4422(12)70259-4 23237904

[B13] Bhattacharyya R. Barren C. Kovacs D. M. (2013). Palmitoylation of amyloid precursor protein regulates amyloidogenic processing in lipid rafts. J. Neurosci. 33, 11169–11183. 10.1523/JNEUROSCI.4704-12.2013 23825420 PMC3718372

[B14] Bolós M. Hu Y. Young K. M. Foa L. Small D. H. (2014). Neurogenin 2 mediates amyloid-β precursor protein-stimulated neurogenesis. J. Biol. Chem. 289, 31253–31261. 10.1074/jbc.M114.581918 25217641 PMC4223326

[B15] Braithwaite S. P. Stock J. B. Lombroso P. J. Nairn A. C. (2012). Protein phosphatases and Alzheimer’s disease. Prog. Mol. Biol. Transl. Sci. 106, 343–379. 10.1016/B978-0-12-396456-4.00012-2 22340724 PMC3739963

[B16] Brouwers N. Sleegers K. Engelborghs S. Bogaerts V. Serneels S. Kamali K.. (2006). Genetic risk and transcriptional variability of amyloid precursor protein in Alzheimer’s disease. Brain 129, 2984–2991. 10.1093/brain/awl212 16931535

[B17] Busciglio J. Gabuzda D. H. Matsudaira P. Yankner B. A. (1993). Generation of β-amyloid in the secretory pathway in neuronal and nonneuronal cells. Proc. Natl. Acad. Sci. U S A 90, 2092–2096. 10.1073/pnas.90.5.2092 8446635 PMC46027

[B18] Buxbaum J. D. Gandy S. E. Cicchetti P. Ehrlich M. E. Czernik A. J. Fracasso R. P.. (1990). Processing of Alzheimer β/A4 amyloid precursor protein: modulation by agents that regulate protein phosphorylation. Proc. Natl. Acad. Sci. U S A 87, 6003–6006. 10.1073/pnas.87.15.6003 2116015 PMC54458

[B19] Buxbaum J. D. Koo E. H. Greengard P. (1993). Protein phosphorylation inhibits production of Alzheimer amyloid β/A4 peptide. Proc. Natl. Acad. Sci. U S A 90, 9195–9198. 10.1073/pnas.90.19.9195 8415676 PMC47529

[B20] Campion D. Dumanchin C. Hannequin D. Dubois B. Belliard S. Puel M.. (1999). Early-onset autosomal dominant Alzheimer disease: prevalence, genetic heterogeneity, and mutation spectrum. Am. J. Hum. Genet. 65, 664–670. 10.1086/302553 10441572 PMC1377972

[B21] Capell A. Beher D. Prokop S. Steiner H. Kaether C. Shearman M. S.. (2005). γ-secretase complex assembly within the early secretory pathway. J. Biol. Chem. 280, 6471–6478. 10.1074/jbc.m409106200 15591316

[B22] Chen F. Hasegawa H. Schmitt-Ulms G. Kawarai T. Bohm C. Katayama T.. (2006). TMP21 is a presenilin complex component that modulates γ-secretase but not epsilon-secretase activity. Nature 440, 1208–1212. 10.1038/nature04667 16641999

[B23] Cheng X. Hart G. W. (2001). Alternative O-glycosylation/O-phosphorylation of serine-16 in murine estrogen receptor β: post-translational regulation of turnover and transactivation activity. J. Biol. Chem. 276, 10570–10575. 10.1074/jbc.M010411200 11150304

[B24] Chou T. Y. Hart G. W. Dang C. V. (1995). c-Myc is glycosylated at threonine 58, a known phosphorylation site and a mutational hot spot in lymphomas. J. Biol. Chem. 270, 18961–18965. 10.1074/jbc.270.32.18961 7642555

[B25] Chun Y. S. Kwon O. H. Oh H. G. Kim T. W. McIntire L. B. Park M. K.. (2015a). Threonine 576 residue of amyloid-β precursor protein regulates its trafficking and processing. Biochem. Biophys. Res. Commun. 467, 955–960. 10.1016/j.bbrc.2015.10.037 26471307

[B26] Chun Y. S. Park Y. Oh H. G. Kim T. W. Yang H. O. Park M. K.. (2015b). O-GlcNAcylation promotes non-amyloidogenic processing of amyloid-β protein precursor via inhibition of endocytosis from the plasma membrane. J. Alzheimers Dis. 44, 261–275. 10.3233/JAD-140096 25208619

[B27] Chyung A. S. Greenberg B. D. Cook D. G. Doms R. W. Lee V. M. (1997). Novel β-secretase cleavage of β-amyloid precursor protein in the endoplasmic reticulum/intermediate compartment of NT2N cells. J. Cell Biol. 138, 671–680. 10.1083/jcb.138.3.671 9245794 PMC2141643

[B28] Colombo A. Bastone A. Ploia C. Sclip A. Salmona M. Forloni G.. (2009). JNK regulates APP cleavage and degradation in a model of Alzheimer’s disease. Neurobiol. Dis. 33, 518–525. 10.1016/j.nbd.2008.12.014 19166938

[B29] Cook D. G. Forman M. S. Sung J. C. Leight S. Kolson D. L. Iwatsubo T.. (1997). Alzheimer’s Aβ(1–42) is generated in the endoplasmic reticulum/intermediate compartment of NT2N cells. Nat. Med. 3, 1021–1023. 10.1038/nm0997-1021 9288730

[B32] Deng Y. Wang Z. Wang R. Zhang X. Zhang S. Wu Y.. (2013). Amyloid-β protein (Aβ) Glu11 is the major β-secretase site of β-site amyloid-β precursor protein-cleaving enzyme 1(BACE1) and shifting the cleavage site to Aβ Asp1 contributes to Alzheimer pathogenesis. Eur. J. Neurosci. 37, 1962–1969. 10.1111/ejn.12235 23773065

[B30] De Strooper B. Annaert W. Cupers P. Saftig P. Craessaerts K. Mumm J. S.. (1999). A presenilin-1-dependent γ-secretase-like protease mediates release of Notch intracellular domain. Nature 398, 518–522. 10.1038/19083 10206645

[B31] De Strooper B. Saftig P. Craessaerts K. Vanderstichele H. Guhde G. Annaert W.. (1998). Deficiency of presenilin-1 inhibits the normal cleavage of amyloid precursor protein. Nature 391, 387–390. 10.1038/34910 9450754

[B33] Dias W. B. Cheung W. D. Wang Z. Hart G. W. (2009). Regulation of calcium/calmodulin-dependent kinase IV by O-GlcNAc modification. J. Biol. Chem. 284, 21327–21337. 10.1074/jbc.M109.007310 19506079 PMC2755857

[B34] Dong Z. Han H. Li H. Bai Y. Wang W. Tu M.. (2015). Long-term potentiation decay and memory loss are mediated by AMPAR endocytosis. J. Clin. Invest. 125, 234–247. 10.1172/JCI77888 25437879 PMC4382266

[B35] Dorval V. Mazzella M. J. Mathews P. M. Hay R. T. Fraser P. E. (2007). Modulation of Aβ generation by small ubiquitin-like modifiers does not require conjugation to target proteins. Biochem. J. 404, 309–316. 10.1042/bj20061451 17346237 PMC1868795

[B36] El Ayadi A. Stieren E. S. Barral J. M. Boehning D. (2012). Ubiquilin-1 regulates amyloid precursor protein maturation and degradation by stimulating K63-linked polyubiquitination of lysine 688. Proc. Natl. Acad. Sci. U S A 109, 13416–13421. 10.1073/pnas.1206786109 22847417 PMC3421158

[B37] Fanutza T. Del Prete D. Ford M. J. Castillo P. E. D’Adamio L. (2015). APP and APLP2 interact with the synaptic release machinery and facilitate transmitter release at hippocampal synapses. Elife 4:e09743. 10.7554/eLife.09743 26551565 PMC4755753

[B38] Ferrer I. Barrachina M. Puig B. Martínez de Lagran M. Martí E. Avila J.. (2005). Constitutive Dyrk1A is abnormally expressed in Alzheimer disease, Down syndrome, Pick disease, and related transgenic models. Neurobiol. Dis. 20, 392–400. 10.1016/j.nbd.2005.03.020 16242644

[B39] Feyt C. Pierrot N. Tasiaux B. Van Hees J. Kienlen-Campard P. Courtoy P. J.. (2007). Phosphorylation of APP695 at Thr668 decreases γ-cleavage and extracellular Aβ. Biochem. Biophys. Res. Commun. 357, 1004–1010. 10.1016/j.bbrc.2007.04.036 17459339

[B40] Gandy S. Czernik A. J. Greengard P. (1988). Phosphorylation of Alzheimer disease amyloid precursor peptide by protein kinase C and Ca^2+^/calmodulin-dependent protein kinase II. Proc. Natl. Acad. Sci. U S A 85, 6218–6221. 10.1073/pnas.85.16.6218 3137567 PMC281937

[B41] Glenner G. G. Wong C. W. (1984). Alzheimer’s disease: initial report of the purification and characterization of a novel cerebrovascular amyloid protein. Biochem. Biophys. Res. Commun. 120, 885–890. 10.1016/s0006-291x(84)80190-4 6375662

[B42] Godyń J. Jończyk J. Panek D. Malawska B. (2016). Therapeutic strategies for Alzheimer’s disease in clinical trials. Pharmacol. Rep. 68, 127–138. 10.1016/j.pharep.2015.07.006 26721364

[B43] Golde T. E. Estus S. Younkin L. H. Selkoe D. J. Younkin S. G. (1992). Processing of the amyloid protein precursor to potentially amyloidogenic derivatives. Science 255, 728–730. 10.1126/science.1738847 1738847

[B44] Goldgaber D. Lerman M. I. McBride O. W. Saffiotti U. Gajdusek D. C. (1987). Characterization and chromosomal localization of a cDNA encoding brain amyloid of Alzheimer’s disease. Science 235, 877–880. 10.1126/science.3810169 3810169

[B45] Greenfield J. P. Tsai J. Gouras G. K. Hai B. Thinakaran G. Checler F.. (1999). Endoplasmic reticulum and trans-Golgi network generate distinct populations of Alzheimer β-amyloid peptides. Proc. Natl. Acad. Sci. U S A 96, 742–747. 10.1073/pnas.96.2.742 9892704 PMC15207

[B46] Griffith L. S. Mathes M. Schmitz B. (1995). β-amyloid precursor protein is modified with O-linked N-acetylglucosamine. J. Neurosci. Res. 41, 270–278. 10.1002/jnr.490410214 7650762

[B47] Haass C. Koo E. H. Mellon A. Hung A. Y. Selkoe D. J. (1992). Targeting of cell-surface β-amyloid precursor protein to lysosomes: alternative processing into amyloid-bearing fragments. Nature 357, 500–503. 10.1038/357500a0 1608449

[B48] Haass C. Lemere C. A. Capell A. Citron M. Seubert P. Schenk D.. (1995). The Swedish mutation causes early-onset Alzheimer’s disease by β-secretase cleavage within the secretory pathway. Nat. Med. 1, 1291–1296. 10.1038/nm1295-1291 7489411

[B49] Halim A. Brinkmalm G. Rüetschi U. Westman-Brinkmalm A. Portelius E. Zetterberg H.. (2011). Site-specific characterization of threonine, serine, and tyrosine glycosylations of amyloid precursor protein/amyloid β-peptides in human cerebrospinal fluid. Proc. Natl. Acad. Sci. U S A 108, 11848–11853. 10.1073/pnas.1102664108 21712440 PMC3141957

[B50] Hardy J. A. Higgins G. A. (1992). Alzheimer’s disease: the amyloid cascade hypothesis. Science 256, 184–185. 10.1126/science.1566067 1566067

[B51] Hart G. W. Slawson C. Ramirez-Correa G. Lagerlof O. (2011). Cross talk between O-GlcNAcylation and phosphorylation: roles in signaling, transcription and chronic disease. Annu. Rev. Biochem. 80, 825–858. 10.1146/annurev-biochem-060608-102511 21391816 PMC3294376

[B52] Hartmann T. Bieger S. C. Bruhl B. Tienari P. J. Ida N. Allsop D.. (1997). Distinct sites of intracellular production for Alzheimer’s disease A β40/42 amyloid peptides. Nat. Med. 3, 1016–1020. 10.1038/nm0997-1016 9288729

[B53] Higashi S. Miyazaki K. (2003). Novel processing of β-amyloid precursor protein catalyzed by membrane type 1 matrix metalloproteinase releases a fragment lacking the inhibitor domain against gelatinase A. Biochemistry 42, 6514–6526. 10.1021/bi020643m 12767235

[B54] Hiltunen M. Lu A. Thomas A. V. Romano D. M. Kim M. Jones P. B.. (2006). Ubiquilin 1 modulates amyloid precursor protein trafficking and Aβ secretion. J. Biol. Chem. 281, 32240–32253. 10.1074/jbc.M603106200 16945923

[B55] Hort J. O’Brien J. T. Gainotti G. Pirttila T. Popescu B. O. Rektorova I.. (2010). EFNS guidelines for the diagnosis and management of Alzheimer’s disease. Eur. J. Neurol. 17, 1236–1248. 10.1111/j.1468-1331.2010.03040.x 20831773

[B58] Hung S. Y. Fu W. M. (2017). Drug candidates in clinical trials for Alzheimer’s disease. J. Biomed. Sci. 24:47. 10.1186/s12929-017-0355-7 28720101 PMC5516350

[B57] Hung A. Y. Haass C. Nitsch R. M. Qiu W. Q. Citron M. Wurtman R. J.. (1993). Activation of protein kinase C inhibits cellular production of the amyloid β-protein. J. Biol. Chem. 268, 22959–22962. 8226807

[B56] Hung A. Y. Selkoe D. J. (1994). Selective ectodomain phosphorylation and regulated cleavage of β-amyloid precursor protein. EMBO J. 13, 534–542. 8313898 10.1002/j.1460-2075.1994.tb06291.x PMC394842

[B59] Huse J. T. Liu K. Pijak D. S. Carlin D. Lee V. M. Doms R. W. (2002). β-secretase processing in the trans-Golgi network preferentially generates truncated amyloid species that accumulate in Alzheimer’s disease brain. J. Biol. Chem. 277, 16278–16284. 10.1074/jbc.M111141200 11847218

[B60] Iijima K. Ando K. Takeda S. Satoh Y. Seki T. Itohara S.. (2000). Neuron-specific phosphorylation of Alzheimer’s β-amyloid precursor protein by cyclin-dependent kinase 5. J. Neurochem. 75, 1085–1091. 10.1046/j.1471-4159.2000.0751085.x 10936190

[B61] Isohara T. Horiuchi A. Watanabe T. Ando K. Czernik A. J. Uno I.. (1999). Phosphorylation of the cytoplasmic domain of Alzheimer’s β-amyloid precursor protein at Ser655 by a novel protein kinase. Biochem. Biophys. Res. Commun. 258, 300–305. 10.1006/bbrc.1999.0637 10329382

[B62] Jacobsen K. T. Iverfeldt K. (2011). O-GlcNAcylation increases non-amyloidogenic processing of the amyloid-β precursor protein (APP). Biochem. Biophys. Res. Commun. 404, 882–886. 10.1016/j.bbrc.2010.12.080 21182826

[B63] Jahangir Z. Ahmad W. Shabbiri K. (2014). Alternate phosphorylation/O-GlcNAc modification on human insulin IRSs: a road towards impaired insulin signaling in Alzheimer and diabetes. Adv. Bioinformatics 2014:324753. 10.1155/2014/324753 25580119 PMC4281456

[B64] Joshi G. Wang Y. (2015). Golgi defects enhance APP amyloidogenic processing in Alzheimer’s disease. Bioessays 37, 240–247. 10.1002/bies.201400116 25546412 PMC4407201

[B65] Judge M. Hornbeck L. Potter H. Padmanabhan J. (2011). Mitosis-specific phosphorylation of amyloid precursor protein at threonine 668 leads to its altered processing and association with centrosomes. Mol. Neurodegener. 6:80. 10.1186/1750-1326-6-80 22112898 PMC3284477

[B66] Kaether C. Schmitt S. Willem M. Haass C. (2006). Amyloid precursor protein and Notch intracellular domains are generated after transport of their precursors to the cell surface. Traffic 7, 408–415. 10.1111/j.1600-0854.2006.00396.x 16536739

[B67] Kanatsu K. Morohashi Y. Suzuki M. Kuroda H. Watanabe T. Tomita T.. (2014). Decreased CALM expression reduces Aβ42 to total Aβ ratio through clathrin-mediated endocytosis of γ-secretase. Nat. Commun. 5:3386. 10.1038/ncomms4386 24577224

[B68] Kaneko M. Koike H. Saito R. Kitamura Y. Okuma Y. Nomura Y. (2010). Loss of HRD1-mediated protein degradation causes amyloid precursor protein accumulation and amyloid-β generation. J. Neurosci. 30, 3924–3932. 10.1523/JNEUROSCI.2422-09.2010 20237263 PMC6632277

[B70] Kang J. Lemaire H. G. Unterbeck A. Salbaum J. M. Masters C. L. Grzeschik K. H.. (1987). The precursor of Alzheimer’s disease amyloid A4 protein resembles a cell-surface receptor. Nature 325, 733–736. 10.1038/325733a0 2881207

[B69] Kang J. Müller-Hill B. (1990). Differential splicing of Alzheimer’s disease amyloid A4 precursor RNA in rat tissues: PreA4_695_ mRNA is predominantly produced in rat and human brain. Biochem. Biophys. Res. Commun. 166, 1192–1200. 10.1016/0006-291x(90)90992-v 1689572

[B71] Kang Y. Zhang Y. Feng Z. Liu M. Li Y. Yang H.. (2017). Nutritional deficiency in early life facilitates aging-associated cognitive decline. Curr. Alzheimer Res. 14, 841–849. 10.2174/1567205014666170425112331 28443508

[B72] Karran E. Mercken M. De Strooper B. (2011). The amyloid cascade hypothesis for Alzheimer’s disease: an appraisal for the development of therapeutics. Nat. Rev. Drug. Discov. 10, 698–712. 10.1038/nrd3505 21852788

[B73] Kasuga K. Shimohata T. Nishimura A. Shiga A. Mizuguchi T. Tokunaga J.. (2009). Identification of independent APP locus duplication in Japanese patients with early-onset alzheimer disease. J. Neurol. Neurosurg. Psychiatry 80, 1050–1052. 10.1136/jnnp.2008.161703 19684239

[B74] Kim B. M. You M.-H. Chen C.-H. Suh J. Tanzi R. E. Ho Lee T. (2016). Inhibition of death-associated protein kinase 1 attenuates the phosphorylation and amyloidogenic processing of amyloid precursor protein. Hum. Mol. Genet. 25, 2498–2513. 10.1093/hmg/ddw114 27094130 PMC6086563

[B75] Kimura A. Hata S. Suzuki T. (2016). Alternative selection of β-site APP-cleaving enzyme 1 (BACE1) cleavage sites in amyloid β-protein precursor (APP) harboring protective and pathogenic mutations within the Aβ sequence. J. Biol. Chem. 291, 24041–24053. 10.1074/jbc.M116.744722 27687728 PMC5104930

[B76] Knops J. Gandy S. Greengard P. Lieberburg I. Sinha S. (1993). Serine phosphorylation of the secreted extracellular domain of APP. Biochem. Biophys. Res. Commun. 197, 380–385. 10.1006/bbrc.1993.2490 8267571

[B77] Koo E. H. Squazzo S. L. (1994). Evidence that production and release of amyloid β-protein involves the endocytic pathway. J. Biol. Chem. 269, 17386–17389. 8021238

[B78] Koo E. H. Squazzo S. L. Selkoe D. J. Koo C. H. (1996). Trafficking of cell-surface amyloid β-protein precursor. I. Secretion, endocytosis and recycling as detected by labeled monoclonal antibody. J. Cell Sci. 109, 991–998. 8743946 10.1242/jcs.109.5.991

[B79] Korvatska O. Leverenz J. B. Jayadev S. McMillan P. Kurtz I. Guo X.. (2015). R47H variant of *TREM2* associated with Alzheimer disease in a large late-onset family: clinical, genetic, and neuropathological study. JAMA Neurol. 72, 920–927. 10.1001/jamaneurol.2015.0979 26076170 PMC4825672

[B80] Lamb B. T. Sisodia S. S. Lawler A. M. Slunt H. H. Kitt C. A. Kearns W. G.. (1993). Introduction and expression of the 400 kilobase *amyloid precursor protein* gene in transgenic mice. Nat. Genet. 5, 22–30. 10.1038/ng0993-22 8220418

[B81] Lee M. S. Kao S. C. Lemere C. A. Xia W. Tseng H. C. Zhou Y.. (2003). APP processing is regulated by cytoplasmic phosphorylation. J. Cell Biol. 163, 83–95. 10.1083/jcb.200301115 14557249 PMC2173445

[B82] Levy-Lahad E. Wasco W. Poorkaj P. Romano D. M. Oshima J. Pettingell W. H.. (1995a). Candidate gene for the chromosome 1 familial Alzheimer’s disease locus. Science 269, 973–977. 10.1126/science.7638622 7638622

[B83] Levy-Lahad E. Wijsman E. M. Nemens E. Anderson L. Goddard K. A. Weber J. L.. (1995b). A familial Alzheimer’s disease locus on chromosome 1. Science 269, 970–973. 10.1126/science.7638621 7638621

[B84] Li J. Ma J. Potter H. (1995). Identification and expression analysis of a potential familial Alzheimer disease gene on chromosome 1 related to AD3. Proc. Natl. Acad. Sci. U S A 92, 12180–12184. 10.1073/pnas.92.26.12180 8618867 PMC40320

[B85] Liebelt F. Vertegaal A. C. (2016). Ubiquitin-dependent and independent roles of SUMO in proteostasis. Am. J. Physiol. Cell Physiol. 311, C284–C296. 10.1152/ajpcell.00091.2016 27335169 PMC5129774

[B87] Liu K. Doms R. W. Lee V. M. (2002). Glu11 site cleavage and N-terminally truncated Aβ production upon BACE overexpression. Biochemistry 41, 3128–3136. 10.1021/bi015800g 11863452

[B86] Liu F. Shi J. Tanimukai H. Gu J. Grundke-Iqbal I. Iqbal K.. (2009). Reduced O-GlcNAcylation links lower brain glucose metabolism and tau pathology in Alzheimer’s disease. Brain 132, 1820–1832. 10.1093/brain/awp099 19451179 PMC2702834

[B88] Liu X. Wang Z. Wu Y. Wang J. Song W. (2013). BACE2 degradation mediated by the macroautophagy-lysosome pathway. Eur. J. Neurosci. 37, 1970–1977. 10.1111/ejn.12204 23773066

[B89] Long J. M. Ray B. Lahiri D. K. (2012). MicroRNA-153 physiologically inhibits expression of amyloid-β precursor protein in cultured human fetal brain cells and is dysregulated in a subset of Alzheimer disease patients. J. Biol. Chem. 287, 31298–31310. 10.1074/jbc.M112.366336 22733824 PMC3438960

[B90] Luo H. B. Xia Y. Y. Shu X. J. Liu Z. C. Feng Y. Liu X. H.. (2014). SUMOylation at K340 inhibits tau degradation through deregulating its phosphorylation and ubiquitination. Proc. Natl. Acad. Sci. U S A 111, 16586–16591. 10.1073/pnas.1417548111 25378699 PMC4246270

[B91] Ly P. T. Wu Y. Zou H. Wang R. Zhou W. Kinoshita A.. (2013). Inhibition of GSK3β-mediated BACE1 expression reduces Alzheimer-associated phenotypes. J. Clin. Invest. 123, 224–235. 10.1172/JCI64516 23202730 PMC3533290

[B92] Maltese W. A. Wilson S. Tan Y. Suomensaari S. Sinha S. Barbour R.. (2001). Retention of the Alzheimer’s amyloid precursor fragment C99 in the endoplasmic reticulum prevents formation of amyloid β-peptide. J. Biol. Chem. 276, 20267–20279. 10.1074/jbc.M007238200 11278337

[B93] Marcinkiewicz M. Seidah N. G. (2000). Coordinated expression of β-amyloid precursor protein and the putative β-secretase BACE and α-secretase ADAM10 in mouse and human brain. J. Neurochem. 75, 2133–2143. 10.1046/j.1471-4159.2000.0752133.x 11032903

[B94] Matsushima T. Saito Y. Elliott J. I. Iijima-Ando K. Nishimura M. Kimura N.. (2012). Membrane-microdomain localization of amyloid β-precursor protein (APP) C-terminal fragments is regulated by phosphorylation of the cytoplasmic Thr668 residue. J. Biol. Chem. 287, 19715–19724. 10.1074/jbc.M111.334847 22511769 PMC3366005

[B95] Mazzitelli S. Xu P. Ferrer I. Davis R. J. Tournier C. (2011). The loss of c-Jun N-terminal protein kinase activity prevents the amyloidogenic cleavage of amyloid precursor protein and the formation of amyloid plaques *in vivo*. J. Neurosci. 31, 16969–16976. 10.1523/JNEUROSCI.4491-11.2011 22114267 PMC6623849

[B96] McFarlane I. Breen K. C. Di Giamberardino L. Moya K. L. (2000). Inhibition of N-glycan processing alters axonal transport of synaptic glycoproteins *in vivo*. Neuroreport 11, 1543–1547. 10.1097/00001756-200005150-00036 10841374

[B97] McFarlane I. Georgopoulou N. Coughlan C. M. Gillian A. M. Breen K. C. (1999). The role of the protein glycosylation state in the control of cellular transport of the amyloid β precursor protein. Neuroscience 90, 15–25. 10.1016/s0306-4522(98)00361-3 10188930

[B98] McKhann G. M. Knopman D. S. Chertkow H. Hyman B. T. Jack C. R. Jr. Kawas C. H.. (2011). The diagnosis of dementia due to Alzheimer’s disease: recommendations from the national institute on aging-Alzheimer’s association workgroups on diagnostic guidelines for Alzheimer’s disease. Alzheimers Dement. 7, 263–269. 10.1016/j.jalz.2011.04.001 21514250 PMC3312024

[B99] Milosch N. Tanriöver G. Kundu A. Rami A. Francois J. C. Baumkötter F.. (2014). Holo-APP and G-protein-mediated signaling are required for sAPPα-induced activation of the Akt survival pathway. Cell Death Dis. 5:e1391. 10.1038/cddis.2014.352 25165877 PMC4454324

[B100] Morel E. Chamoun Z. Lasiecka Z. M. Chan R. B. Williamson R. L. Vetanovetz C.. (2013). Phosphatidylinositol-3-phosphate regulates sorting and processing of amyloid precursor protein through the endosomal system. Nat. Commun. 4:2250. 10.1038/ncomms3250 23907271 PMC3905799

[B101] Mullan M. Houlden H. Windelspecht M. Fidani L. Lombardi C. Diaz P.. (1992). A locus for familial early-onset Alzheimer’s disease on the long arm of chromosome 14, proximal to the α 1-antichymotrypsin gene. Nat. Genet. 2, 340–342. 10.1038/ng1292-340 1303291

[B102] Munger J. S. Haass C. Lemere C. A. Shi G. P. Wong W. S. Teplow D. B.. (1995). Lysosomal processing of amyloid precursor protein to A beta peptides: a distinct role for cathepsin S. Biochem. J. 311, 299–305. 10.1042/bj3110299 7575468 PMC1136152

[B103] Muresan V. Ladescu Muresan Z. (2015). Amyloid-β precursor protein: multiple fragments, numerous transport routes and mechanisms. Exp. Cell Res. 334, 45–53. 10.1016/j.yexcr.2014.12.014 25573596 PMC4433838

[B104] Muresan V. Muresan Z. (2012). A persistent stress response to impeded axonal transport leads to accumulation of amyloid-β in the endoplasmic reticulum and is a probable cause of sporadic Alzheimer’s disease. Neurodegener. Dis. 10, 60–63. 10.1159/000332815 22156573 PMC3363352

[B105] Neve R. L. Finch E. A. Dawes L. R. (1988). Expression of the Alzheimer amyloid precursor gene transcripts in the human brain. Neuron 1, 669–677. 10.1016/0896-6273(88)90166-3 2908447

[B106] Oishi M. Nairn A. C. Czernik A. J. Lim G. S. Isohara T. Gandy S. E.. (1997). The cytoplasmic domain of Alzheimer’s amyloid precursor protein is phosphorylated at Thr654, Ser655, and Thr668 in adult rat brain and cultured cells. Mol. Med. 3, 111–123. 9085254 PMC2230054

[B107] Oliveira J. M. Henriques A. G. Martins F. Rebelo S. da Cruz e Silva O. A. (2015). Amyloid-β modulates both AβPP and tau phosphorylation. J. Alzheimers Dis. 45, 495–507. 10.3233/JAD-142664 25589714

[B108] Pahlsson P. Shakin-Eshleman S. H. Spitalnik S. L. (1992). N-linked glycosylation of β-amyloid precursor protein. Biochem. Biophys. Res. Commun. 189, 1667–1673. 10.1016/0006-291X(92)90269-Q 1482372

[B109] Pei J. J. Braak E. Braak H. Grundke-Iqbal I. Iqbal K. Winblad B.. (1999). Distribution of active glycogen synthase kinase 3β (GSK-3β) in brains staged for Alzheimer disease neurofibrillary changes. J. Neuropathol. Exp. Neurol. 58, 1010–1019. 10.1097/00005072-199909000-00011 10499443

[B110] Perdivara I. Petrovich R. Allinquant B. Deterding L. J. Tomer K. B. Przybylski M. (2009). Elucidation of O-glycosylation structures of the β-amyloid precursor protein by liquid chromatography-mass spectrometry using electron transfer dissociation and collision induced dissociation. J. Proteome Res. 8, 631–642. 10.1021/pr800758g 19093876 PMC2743936

[B111] Placido A. I. Pereira C. M. Duarte A. I. Candeias E. Correia S. C. Santos R. X.. (2014). The role of endoplasmic reticulum in amyloid precursor protein processing and trafficking: implications for Alzheimer’s disease. Biochim. Biophys. Acta 1842, 1444–1453. 10.1016/j.bbadis.2014.05.003 24832819

[B112] Qing H. He G. Ly P. T. Fox C. J. Staufenbiel M. Cai F.. (2008). Valproic acid inhibits Aβ production, neuritic plaque formation, and behavioral deficits in Alzheimer’s disease mouse models. J. Exp. Med. 205, 2781–2789. 10.1084/jem.20081588 18955571 PMC2585842

[B113] Qiu C. Kivipelto M. von Strauss E. (2009). Epidemiology of Alzheimer’s disease: occurrence, determinants, and strategies toward intervention. Dialogues Clin. Neurosci. 11, 111–128. 19585947 10.31887/DCNS.2009.11.2/cqiu PMC3181909

[B114] Rebelo S. Vieira S. I. Esselmann H. Wiltfang J. da Cruz e Silva E. F. da Cruz e Silva O. A. (2007). Tyrosine 687 phosphorylated Alzheimer’s amyloid precursor protein is retained intracellularly and exhibits a decreased turnover rate. Neurodegener. Dis. 4, 78–87. 10.1159/000101831 17596701

[B115] Robakis N. K. Ramakrishna N. Wolfe G. Wisniewski H. M. (1987). Molecular cloning and characterization of a cDNA encoding the cerebrovascular and the neuritic plaque amyloid peptides. Proc. Natl. Acad. Sci. U S A 84, 4190–4194. 10.1073/pnas.84.12.4190 3035574 PMC305050

[B116] Rogaev E. I. Sherrington R. Rogaeva E. A. Levesque G. Ikeda M. Liang Y.. (1995). Familial Alzheimer’s disease in kindreds with missense mutations in a gene on chromosome 1 related to the Alzheimer’s disease type 3 gene. Nature 376, 775–778. 10.1038/376775a0 7651536

[B117] Rovelet-Lecrux A. Frebourg T. Tuominen H. Majamaa K. Campion D. Remes A. M. (2007). APP locus duplication in a Finnish family with dementia and intracerebral haemorrhage. J. Neurol. Neurosurg. Psychiatry 78, 1158–1159. 10.1136/jnnp.2006.113514 17442758 PMC2117532

[B118] Rovelet-Lecrux A. Hannequin D. Raux G. Le Meur N. Laquerrière A. Vital A.. (2006). APP locus duplication causes autosomal dominant early-onset alzheimer disease with cerebral amyloid angiopathy. Nat. Genet. 38, 24–26. 10.1038/ng1718 16369530

[B119] Ruan H. B. Nie Y. Yang X. (2013). Regulation of protein degradation by O-GlcNAcylation: crosstalk with ubiquitination. Mol. Cell Proteomics 12, 3489–3497. 10.1074/mcp.R113.029751 23824911 PMC3861702

[B120] Ryoo S. R. Cho H. J. Lee H. W. Jeong H. K. Radnaabazar C. Kim Y. S.. (2008). Dual-specificity tyrosine(Y)-phosphorylation regulated kinase 1A-mediated phosphorylation of amyloid precursor protein: evidence for a functional link between Down syndrome and Alzheimer’s disease. J. Neurochem. 104, 1333–1344. 10.1111/j.1471-4159.2007.05075.x 18005339

[B121] Ryoo S. R. Jeong H. K. Radnaabazar C. Yoo J. J. Cho H. J. Lee H. W.. (2007). DYRK1A-mediated hyperphosphorylation of Tau. A functional link between Down syndrome and Alzheimer disease. J. Biol. Chem. 282, 34850–34857. 10.1074/jbc.M707358200 17906291

[B122] Schellenberg G. D. Bird T. D. Wijsman E. M. Orr H. T. Anderson L. Nemens E.. (1992). Genetic linkage evidence for a familial Alzheimer’s disease locus on chromosome 14. Science 258, 668–671. 10.1126/science.1411576 1411576

[B123] Segal-Gidan F. Cherry D. Jones R. Williams B. Hewett L. Chodosh J. (2011). Alzheimer’s disease management guideline: update 2008. Alzheimers Dement. 7, e51–e59. 10.1016/j.jalz.2010.07.005 21546322

[B124] Sherrington R. Rogaev E. I. Liang Y. Rogaeva E. A. Levesque G. Ikeda M.. (1995). Cloning of a gene bearing missense mutations in early-onset familial Alzheimer’s disease. Nature 375, 754–760. 10.1038/375754a0 7596406

[B125] Sisodia S. S. (1992). β-amyloid precursor protein cleavage by a membrane-bound protease. Proc. Natl. Acad. Sci. U S A 89, 6075–6079. 10.1073/pnas.89.13.6075 1631093 PMC49440

[B126] Sisodia S. S. Koo E. H. Hoffman P. N. Perry G. Price D. L. (1993). Identification and transport of full-length amyloid precursor proteins in rat peripheral nervous system. J. Neurosci. 13, 3136–3142. 8331390 10.1523/JNEUROSCI.13-07-03136.1993 PMC6576678

[B127] Skovronsky D. M. Moore D. B. Milla M. E. Doms R. W. Lee V. M. (2000). Protein kinase C-dependent α-secretase competes with β-secretase for cleavage of amyloid-β precursor protein in the trans-golgi network. J. Biol. Chem. 275, 2568–2575. 10.1074/jbc.275.4.2568 10644715

[B128] Sleegers K. Brouwers N. Gijselinck I. Theuns J. Goossens D. Wauters J.. (2006). APP duplication is sufficient to cause early onset alzheimer’s dementia with cerebral amyloid angiopathy. Brain 129, 2977–2983. 10.1093/brain/awl203 16921174

[B129] Song W. Nadeau P. Yuan M. Yang X. Shen J. Yankner B. A. (1999). Proteolytic release and nuclear translocation of Notch-1 are induced by presenilin-1 and impaired by pathogenic presenilin-1 mutations. Proc. Natl. Acad. Sci. U S A 96, 6959–6963. 10.1073/pnas.96.12.6959 10359821 PMC22024

[B131] Song W. J. Song E. A. Jung M. S. Choi S. H. Baik H. H. Jin B. K.. (2015). Phosphorylation and inactivation of glycogen synthase kinase 3β (GSK3β) by dual-specificity tyrosine phosphorylation-regulated kinase 1A (Dyrk1A). J. Biol. Chem. 290, 2321–2333. 10.1074/jbc.M114.594952 25477508 PMC4303684

[B134] Standen C. L. Brownlees J. Grierson A. J. Kesavapany S. Lau K. F. McLoughlin D. M.. (2001). Phosphorylation of thr(668) in the cytoplasmic domain of the Alzheimer’s disease amyloid precursor protein by stress-activated protein kinase 1b (Jun N-terminal kinase-3). J. Neurochem. 76, 316–320. 10.1046/j.1471-4159.2001.00102.x 11146006

[B135] Stephens D. J. Austen B. M. (1996). Metabolites of the β-amyloid precursor protein generated by β-secretase localise to the trans-Golgi network and late endosome in 293 cells. J. Neurosci. Res. 46, 211–225. 10.1002/(SICI)1097-4547(19961015)46:2<211::AID-JNR9>3.0.CO;2-K 8915898

[B132] St. George-Hyslop P. Haines J. Rogaev E. Mortilla M. Vaula G. Pericak-Vance M.. (1992). Genetic evidence for a novel familial Alzheimer’s disease locus on chromosome 14. Nat. Genet. 2, 330–334. 10.1038/ng1292-330 1303289

[B133] St. George-Hyslop P. H. Tanzi R. E. Polinsky R. J. Haines J. L. Nee L. Watkins P. C.. (1987). The genetic defect causing familial Alzheimer’s disease maps on chromosome 21. Science 235, 885–890. 10.1126/science.2880399 2880399

[B136] Sun X. Bromley-Brits K. Song W. (2012). Regulation of β-site APP-cleaving enzyme 1 gene expression and its role in Alzheimer’s disease. J. Neurochem. 120, 62–70. 10.1111/j.1471-4159.2011.07515.x 22122349

[B138] Sun X. He G. Qing H. Zhou W. Dobie F. Cai F.. (2006a). Hypoxia facilitates Alzheimer’s disease pathogenesis by up-regulating BACE1 gene expression. Proc. Natl. Acad. Sci. U S A 103, 18727–18732. 10.1073/pnas.0606298103 17121991 PMC1693730

[B137] Sun X. He G. Song W. (2006b). BACE2, as a novel APP theta-secretase, is not responsible for the pathogenesis of Alzheimer’s disease in Down syndrome. FASEB J. 20, 1369–1376. 10.1096/fj.05-5632com 16816112

[B139] Sun X. Wang Y. Qing H. Christensen M. A. Liu Y. Zhou W.. (2005). Distinct transcriptional regulation and function of the human *BACE2* and *BACE1* genes. FASEB J. 19, 739–749. 10.1096/fj.04-3426com 15857888

[B140] Sun X. Wu Y. Chen B. Zhang Z. Zhou W. Tong Y.. (2011). Regulator of calcineurin 1 (RCAN1) facilitates neuronal apoptosis through caspase-3 activation. J. Biol. Chem. 286, 9049–9062. 10.1074/jbc.m110.177519 21216952 PMC3059004

[B141] Sun X. Wu Y. Herculano B. Song W. (2014). RCAN1 overexpression exacerbates calcium overloading-induced neuronal apoptosis. PLoS One 9:e95471. 10.1371/journal.pone.0095471 24751678 PMC3994074

[B142] Suzuki T. Nairn A. C. Gandy S. E. Greengard P. (1992). Phosphorylation of Alzheimer amyloid precursor protein by protein kinase C. Neuroscience 48, 755–761. 10.1016/0306-4522(92)90264-3 1630623

[B143] Suzuki T. Oishi M. Marshak D. R. Czernik A. J. Nairn A. C. Greengard P. (1994). Cell cycle-dependent regulation of the phosphorylation and metabolism of the Alzheimer amyloid precursor protein. EMBO J. 13, 1114–1122. 8131745 10.1002/j.1460-2075.1994.tb06360.x PMC394920

[B144] Takahashi K. Niidome T. Akaike A. Kihara T. Sugimoto H. (2008). Phosphorylation of amyloid precursor protein (APP) at Tyr687 regulates APP processing by α- and γ-secretase. Biochem. Biophys. Res. Commun. 377, 544–549. 10.1016/j.bbrc.2008.10.013 18854169

[B145] Takasugi N. Tomita T. Hayashi I. Tsuruoka M. Niimura M. Takahashi Y.. (2003). The role of presenilin cofactors in the γ-secretase complex. Nature 422, 438–441. 10.1038/nature01506 12660785

[B146] Tanimukai H. Grundke-Iqbal I. Iqbal K. (2005). Up-regulation of inhibitors of protein phosphatase-2A in Alzheimer’s disease. Am. J. Pathol. 166, 1761–1771. 10.1016/s0002-9440(10)62486-8 15920161 PMC1602412

[B147] Tanzi R. E. Gusella J. F. Watkins P. C. Bruns G. A. St. George-Hyslop P. Van Keuren M. L.. (1987). Amyloid β protein gene: cDNA, mRNA distribution, and genetic linkage near the Alzheimer locus. Science 235, 880–884. 10.1126/science.2949367 2949367

[B148] Tanzi R. E. McClatchey A. I. Lamperti E. D. Villa-Komaroff L. Gusella J. F. Neve R. L. (1988). Protease inhibitor domain encoded by an amyloid protein precursor mRNA associated with Alzheimer’s disease. Nature 331, 528–530. 10.1038/331528a0 2893290

[B149] Tarr P. E. Contursi C. Roncarati R. Noviello C. Ghersi E. Scheinfeld M. H.. (2002). Evidence for a role of the nerve growth factor receptor TrkA in tyrosine phosphorylation and processing of β-APP. Biochem. Biophys. Res. Commun. 295, 324–329. 10.1016/s0006-291x(02)00678-2 12150951

[B150] Tarrant M. K. Rho H. S. Xie Z. Jiang Y. L. Gross C. Culhane J. C.. (2012). Regulation of CK2 by phosphorylation and O-GlcNAcylation revealed by semisynthesis. Nat. Chem. Biol. 8, 262–269. 10.1038/nchembio.771 22267120 PMC3288285

[B151] Toh W. H. Gleeson P. A. (2016). Dysregulation of intracellular trafficking and endosomal sorting in Alzheimer’s disease: controversies and unanswered questions. Biochem. J. 473, 1977–1993. 10.1042/bcj20160147 27407168

[B152] Tomita S. Kirino Y. Suzuki T. (1998). Cleavage of Alzheimer’s amyloid precursor protein (APP) by secretases occurs after O-glycosylation of APP in the protein secretory pathway. Identification of intracellular compartments in which APP cleavage occurs without using toxic agents that interfere with protein metabolism. J. Biol. Chem. 273, 6277–6284. 10.1074/jbc.273.11.6277 9497354

[B153] Triaca V. Sposato V. Bolasco G. Ciotti M. T. Pelicci P. Bruni A. C.. (2016). NGF controls APP cleavage by downregulating APP phosphorylation at Thr668: relevance for Alzheimer’s disease. Aging Cell 15, 661–672. 10.1111/acel.12473 27076121 PMC4933663

[B154] Vassar R. Bennett B. D. Babu-Khan S. Kahn S. Mendiaz E. A. Denis P.. (1999). β-secretase cleavage of Alzheimer’s amyloid precursor protein by the transmembrane aspartic protease BACE. Science 286, 735–741. 10.1126/science.286.5440.735 10531052

[B155] Vieira S. I. Rebelo S. Esselmann H. Wiltfang J. Lah J. Lane R.. (2010). Retrieval of the Alzheimer’s amyloid precursor protein from the endosome to the TGN is S655 phosphorylation state-dependent and retromer-mediated. Mol. Neurodegener. 5:40. 10.1186/1750-1326-5-40 20937087 PMC2994555

[B156] Vingtdeux V. Hamdane M. Gompel M. Bégard S. Drobecq H. Ghestem A.. (2005). Phosphorylation of amyloid precursor carboxy-terminal fragments enhances their processing by a γ-secretase-dependent mechanism. Neurobiol. Dis. 20, 625–637. 10.1016/j.nbd.2005.05.004 15936948

[B157] Walsh D. M. Selkoe D. J. (2007). Aβ oligomers - a decade of discovery. J. Neurochem. 101, 1172–1184. 10.1111/j.1471-4159.2006.04426.x 17286590

[B158] Walter J. Capell A. Grunberg J. Pesold B. Schindzielorz A. Prior R.. (1996). The Alzheimer’s disease-associated presenilins are differentially phosphorylated proteins located predominantly within the endoplasmic reticulum. Mol. Med. 2, 673–691. 8972483 PMC2230134

[B159] Walter J. Capell A. Hung A. Y. Langen H. Schnölzer M. Thinakaran G.. (1997). Ectodomain phosphorylation of β-amyloid precursor protein at two distinct cellular locations. J. Biol. Chem. 272, 1896–1903. 10.1074/jbc.272.3.1896 8999878

[B161] Wang T. Liu H. Wang Y. Liu C. Sun X. (2014). RCAN1 increases Aβ generation by promoting N-glycosylation via Oligosaccharyltransferase. Curr. Alzheimer Res. 11, 332–339. 10.2174/1567205011666140331225855 24720891

[B160] Wang H.-Y. Pisano M. R. Friedman E. (1994). Attenuated protein kinase C activity and translocation in Alzheimer’s disease brain. Neurobiol. Aging 15, 293–298. 10.1016/0197-4580(94)90023-x 7936052

[B162] Watanabe T. Hikichi Y. Willuweit A. Shintani Y. Horiguchi T. (2012). FBL2 regulates amyloid precursor protein (APP) metabolism by promoting ubiquitination-dependent APP degradation and inhibition of APP endocytosis. J. Neurosci. 32, 3352–3365. 10.1523/jneurosci.5659-11.2012 22399757 PMC6621050

[B163] Wegiel J. Dowjat K. Kaczmarski W. Kuchna I. Nowicki K. Frackowiak J.. (2008). The role of overexpressed DYRK1A protein in the early onset of neurofibrillary degeneration in Down syndrome. Acta Neuropathol. 116, 391–407. 10.1007/s00401-008-0419-6 18696092 PMC2656568

[B164] Weidemann A. König G. Bunke D. Fischer P. Salbaum J. M. Masters C. L.. (1989). Identification, biogenesis, and localization of precursors of Alzheimer’s disease A4 amyloid protein. Cell 57, 115–126. 10.1016/0092-8674(89)90177-3 2649245

[B165] Willem M. Tahirovic S. Busche M. A. Ovsepian S. V. Chafai M. Kootar S.. (2015). η-Secretase processing of APP inhibits neuronal activity in the hippocampus. Nature 526, 443–447. 10.1038/nature14864 26322584 PMC6570618

[B166] Wilson C. A. Doms R. W. Zheng H. Lee V. M. (2002). Presenilins are not required for A β42 production in the early secretory pathway. Nat. Neurosci. 5, 849–855. 10.1038/nn898 12145638

[B169] Wu Y. Deng Y. Zhang S. Luo Y. Cai F. Zhang Z.. (2015). Amyloid-β precursor protein facilitates the regulator of calcineurin 1-mediated apoptosis by downregulating proteasome subunit α type-5 and proteasome subunit β type-7. Neurobiol. Aging 36, 169–177. 10.1016/j.neurobiolaging.2014.07.029 25194880

[B170] Wu Y. Ly P. T. T. Song W. (2014). Aberrant expression of RCAN1 in Alzheimer’s pathogenesis: a new molecular mechanism and a novel drug target. Mol. Neurobiol. 50, 1085–1097. 10.1007/s12035-014-8704-y 24752590

[B167] Wu Y. Song W. (2013). Regulation of RCAN1 translation and its role in oxidative stress-induced apoptosis. FASEB J. 27, 208–221. 10.1096/fj.12-213124 23038757

[B171] Wu Y. Zhang S. Xu Q. Zou H. Zhou W. Cai F.. (2016). Regulation of global gene expression and cell proliferation by APP. Sci. Rep. 6:22460. 10.1038/srep22460 26936520 PMC4776145

[B172] Yamazaki T. Koo E. H. Selkoe D. J. (1996). Trafficking of cell-surface amyloid β-protein precursor. II. Endocytosis, recycling and lysosomal targeting detected by immunolocalization. J. Cell Sci. 109, 999–1008. 8743947 10.1242/jcs.109.5.999

[B173] Yang Y. Song W. (2013). Molecular links between Alzheimer’s disease and diabetes mellitus. Neuroscience 250, 140–150. 10.1016/j.neuroscience.2013.07.009 23867771

[B174] Yang Y. Wu Y. Zhang S. Song W. (2013). High glucose promotes Aβ production by inhibiting APP degradation. PLoS One 8:e69824. 10.1371/journal.pone.0069824 23894546 PMC3720941

[B175] Yazaki M. Tagawa K. Maruyama K. Sorimachi H. Tsuchiya T. Ishiura S.. (1996). Mutation of potential N-linked glycosylation sites in the Alzheimer’s disease amyloid precursor protein (APP). Neurosci. Lett. 221, 57–60. 10.1016/s0304-3940(96)13285-7 9014180

[B176] Yoshikai S. Sasaki H. Doh-ura K. Furuya H. Sakaki Y. (1990). Genomic organization of the human amyloid β-protein precursor gene. Gene 87, 257–263. 10.1016/0378-1119(90)90310-n 2110105

[B177] Zambrano N. Bruni P. Minopoli G. Mosca R. Molino D. Russo C.. (2001). The β-amyloid precursor protein APP is tyrosine-phosphorylated in cells expressing a constitutively active form of the Abl protoncogene. J. Biol. Chem. 276, 19787–19792. 10.1074/jbc.m100792200 11279131

[B178] Zeidan Q. Hart G. W. (2010). The intersections between O-GlcNAcylation and phosphorylation: implications for multiple signaling pathways. J. Cell Sci. 123, 13–22. 10.1242/jcs.053678 20016062 PMC2794709

[B179] Zeng J. Chen L. Wang Z. Chen Q. Fan Z. Jiang H.. (2017). Marginal vitamin A deficiency facilitates Alzheimer’s pathogenesis. Acta Neuropathol. 133, 967–982. 10.1007/s00401-017-1669-y 28130638

[B187] Zhang Z. Nadeau P. Song W. Donoviel D. Yuan M. Bernstein A.. (2000). Presenilins are required for γ-secretase cleavage of β-APP and transmembrane cleavage of Notch-1. Nat. Cell Biol. 2, 463–465. 10.1038/35017108 10878814

[B186] Zhang Y. Q. Sarge K. D. (2008). Sumoylation of amyloid precursor protein negatively regulates Aβ aggregate levels. Biochem. Biophys. Res. Commun. 374, 673–678. 10.1016/j.bbrc.2008.07.109 18675254 PMC2596940

[B182] Zhang X. Song W. (2013). The role of APP and BACE1 trafficking in APP processing and amyloid-β generation. Alzheimers Res. Ther. 5:46. 10.1186/alzrt211 24103387 PMC3978418

[B185] Zhang Y. Song W. (2017). Islet amyloid polypeptide: another key molecule in Alzheimer’s pathogenesis? Prog. Neurobiol. 153, 100–120. 10.1016/j.pneurobio.2017.03.001 28274676

[B180] Zhang S. Wang Z. Cai F. Zhang M. Wu Y. Zhang J.. (2017). BACE1 cleavage site selection critical for amyloidogenesis and Alzheimer’s pathogenesis. J. Neurosci. 37, 6915–6925. 10.1523/jneurosci.0340-17.2017 28626014 PMC6705717

[B183] Zhang X. Wu Y. Cai F. Liu S. Bromley-Brits K. Xia K.. (2017). A novel alzheimer-associated SNP in *Tmp21* increases amyloidogenesis. Mol. Neurobiol. [Epub ahead of print]. 10.1007/s12035-017-0459-9 28233271

[B184] Zhang X. Wu Y. Duan X. Chen W. Zou H. Zhang M.. (2015). Upregulation of SET expression by BACE1 and its implications in Down syndrome. Mol. Neurobiol. 51, 781–790. 10.1007/s12035-014-8782-x 24935721

[B181] Zhang S. Zhang M. Cai F. Song W. (2013). Biological function of Presenilin and its role in AD pathogenesis. Transl. Neurodegener. 2:15. 10.1186/2047-9158-2-15 23866842 PMC3718700

[B188] Zhao G. Mao G. Tan J. Dong Y. Cui M. Z. Kim S. H.. (2004). Identification of a new presenilin-dependent ζ-cleavage site within the transmembrane domain of amyloid precursor protein. J. Biol. Chem. 279, 50647–50650. 10.1074/jbc.c400473200 15485850

[B189] Zimmermann K. Herget T. Salbaum J. M. Schubert W. Hilbich C. Cramer M.. (1988). Localization of the putative precursor of Alzheimer’s disease-specific amyloid at nuclear envelopes of adult human muscle. EMBO J. 7, 367–372. 2896589 10.1002/j.1460-2075.1988.tb02822.x PMC454328

